# Quantifying the potential value of antigen-detection rapid diagnostic tests for COVID-19: a modelling analysis

**DOI:** 10.1186/s12916-021-01948-z

**Published:** 2021-03-09

**Authors:** Saskia Ricks, Emily A. Kendall, David W. Dowdy, Jilian A. Sacks, Samuel G. Schumacher, Nimalan Arinaminpathy

**Affiliations:** 1 MRC Centre for Global Infectious Disease Analysis, Imperial College London, London, UK; 2 Division of Infectious Diseases, Johns Hopkins University School of Medicine, Baltimore, MD USA; 3 Department of Epidemiology, The Johns Hopkins Bloomberg School of Public Health, Baltimore, MD USA; 4 Foundation for Innovative New Diagnostics, Geneva, Switzerland

**Keywords:** Antigen, Rapid diagnostic tests, COVID-19

## Abstract

**Background:**

Testing plays a critical role in treatment and prevention responses to the COVID-19 pandemic. Compared to nucleic acid tests (NATs), antigen-detection rapid diagnostic tests (Ag-RDTs) can be more accessible, but typically have lower sensitivity and specificity. By quantifying these trade-offs, we aimed to inform decisions about when an Ag-RDT would offer greater public health value than reliance on NAT.

**Methods:**

Following an expert consultation, we selected two use cases for analysis: rapid identification of people with COVID-19 amongst patients admitted with respiratory symptoms in a ‘hospital’ setting and early identification and isolation of people with mildly symptomatic COVID-19 in a ‘community’ setting. Using decision analysis, we evaluated the health system cost and health impact (deaths averted and infectious days isolated) of an Ag-RDT-led strategy, compared to a strategy based on NAT and clinical judgement. We adopted a broad range of values for ‘contextual’ parameters relevant to a range of settings, including the availability of NAT and the performance of clinical judgement. We performed a multivariate sensitivity analysis to all of these parameters.

**Results:**

In a hospital setting, an Ag-RDT-led strategy would avert more deaths than a NAT-based strategy, and at lower cost per death averted, when the sensitivity of clinical judgement is less than 90%, and when NAT results are available in time to inform clinical decision-making for less than 85% of patients. The use of an Ag-RDT is robustly supported in community settings, where it would avert more transmission at lower cost than relying on NAT alone, under a wide range of assumptions.

**Conclusions:**

Despite their imperfect sensitivity and specificity, Ag-RDTs have the potential to be simultaneously more impactful, and have a lower cost per death and infectious person-days averted, than current approaches to COVID-19 diagnostic testing.

**Supplementary Information:**

The online version contains supplementary material available at 10.1186/s12916-021-01948-z.

## Background

Virological testing is a critical part of the global response to SARS-CoV-2 [1–3]. Early diagnosis allows infectious cases to be isolated in a timely manner, thus minimising opportunities for transmission. Amongst those at risk of severe outcomes of the disease, early diagnosis and initiation of appropriate therapy can substantially improve outcomes and avert mortality [4–7]. Nucleic acid tests (NATs) have been widely implemented in well-resourced settings since the outset of the pandemic and have the benefit of high sensitivity and specificity for current or recent infection. However, these tests are challenging to implement at scale, particularly in resource-poor settings: they are costly and require good specimen transport systems, laboratory infrastructure, and highly trained technicians. Delays of a week or more in obtaining results after collecting specimens are therefore common [8–10], and in such cases, a NAT result adds little value to decisions around isolation or clinical management.

The emergence of antigen-detection rapid diagnostic tests (Ag-RDTs) may help to address some of these challenges. The World Health Organization (WHO) have recently published Target Product Profiles for such tests [11], which detect SARS-CoV-2 proteins (antigens) to diagnose active infection. Ag-RDTs can be conducted relatively easily, at low cost, and within minutes, at the point of care without need for a laboratory. However, they have lower sensitivity and specificity and may miss SARS-CoV-2 in specimens with lower quantities of virus. For example, available Ag-RDTs were estimated to have less than 80% sensitivity for COVID-19, compared with > 90% for NAT [12]. We therefore sought to quantify these trade-offs between Ag-RDT-based testing and NAT-based testing in the context of resource-limited settings.

## Methods

### Overview

Our primary objective was to identify scenarios in which an Ag-RDT might offer greater individual and public health value at lower cost than reliance on NAT, across a variety of resource-limited settings. To accomplish this, we first defined key use cases and plausible ranges for parameter values, in consultation with a group of experts deeply involved in their country’s response to COVID-19. To identify principles that might generalise across countries, these experts were drawn from a range of different country settings; the ranges in parameter values also served to incorporate variation across these settings. (As described below, a key aim of our analysis was to analyse the most important sources of variation.) We then constructed decision trees that included both costs to the health system (e.g. treatment and management of hospitalised cases) and relevant health outcomes (deaths and infectious person-days averted). Finally, we simulated overall costs and outcomes under a wide array of parameter values and compared testing strategies using Ag-RDTs, with those using NAT where available. We also constructed a user-friendly online tool that enables public health practitioners to examine model outputs for input parameter values relevant to their own settings [13].

#### Model scenarios and structure

**Fig. 1:**
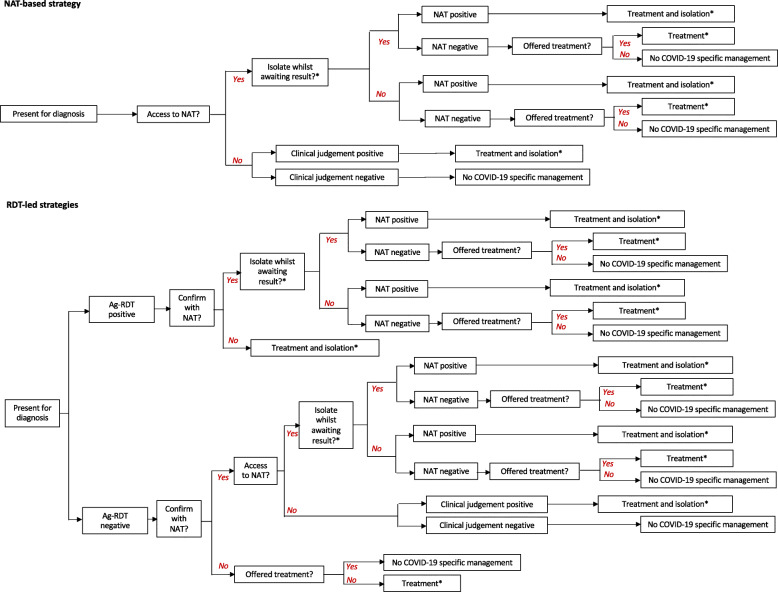
Schematic illustration of the decision tree approach. As described in the main text, our analysis focuses on the direct benefit to patients being tested in different settings. *In the hospital setting, we assumed that all patients were provided with supportive care (e.g. oxygen support) regardless of test results, as such care would be provided based on symptoms and not aetiology. However, we modelled the use of the test in guiding decisions about whom to isolate and to treat with dexamethasone. Treatment did not apply to the community setting. Costs and deaths/infectious days averted were accumulated along each branch of the diagram as appropriate (for example, counting the cost of interim isolation along any branch labelled ‘Yes’ following ‘Isolate whilst awaiting result?’)

We denote an ‘Ag-RDT-led strategy’ as any testing strategy in which an Ag-RDT is the first diagnostic test performed (with the potential for follow-up NAT confirmation). As an illustrative example, we focused on an Ag-RDT with sensitivity and specificity of 80% and 98% respectively, relative to NAT, and costing 5 US$ per test, consistent with recent WHO interim guidance and antigen-detection tests authorised for emergency use by the United States Food and Drug Administration (FDA) [14, 15]. We compared the impact of using an Ag-RDT-led strategy to that of a ‘NAT-based strategy’ in which NAT was the only virological test performed, with reliance on clinical judgement where sufficiently rapid NAT results were not available (see Fig. 1 for a summary of the diagnostic strategies modelled). To inform relevant use case scenarios, we consulted experts from India, South Africa, Nigeria, and Brazil to elicit expert opinion on the ways in which Ag-RDTs could offer value in their own country settings (Additional file 1: Text S1). Based on this input, we selected two use case scenarios, as listed in Table 1: (i) a ‘hospital setting’, where the test is used to support infection control and treatment decisions amongst patients being admitted to hospital with respiratory symptoms and (ii) a ‘community’ setting, where the test is used in decentralised community clinics to identify cases of COVID-19 who should self-isolate. Although Ag-RDTs could also be considered for use in identifying asymptomatic infections, both of these focal scenarios involved testing of only symptomatic individuals.

**Table 1:** The use cases included in the present analysis

Use case scenario	Description	Assumed prevalence (%)	Purpose of testing
Hospital setting	Testing amongst all patients being hospitalised with respiratory symptoms	25	To identify patients with COVID-19 who should be housed in isolation wards (reducing infection risk in hospital) Amongst admissions with severe symptoms, to identify those with COVID-19 who might benefit from anti-inflammatory treatment (to reduce deaths)
Community setting	Decentralised, community-level facility available to all individuals with symptoms who want to be tested for COVID-19	5	To identify COVID-19 amongst people with mild COVID-consistent symptoms. Positive test results would trigger isolation and contact tracing, to minimise opportunities for transmission.

We performed sensitivity analysis on the assumed prevalence, varying the hospital setting prevalence between 10 and 30% and community setting prevalence between 1 and 10%. Results are presented Additional file 7: Fig.S4

For both use cases we constructed decision trees (Fig. 1; Additional file 2: Text S2) that represent the diagnostic use of the Ag-RDT, actions taken in response to the test results (or lack of results), and resulting outcomes. For simplicity and transparency, this model does not incorporate transmission dynamics but approximates epidemiological benefits based on the incremental change in the number of days that infectious individuals spend out of isolation; the magnitude of downstream impact would depend on factors, such as the rate of epidemic growth and the contact patterns of symptomatic versus pre- or asymptomatic cases, that are not specified in our model. Our focus is therefore on the direct benefits that would accrue to patients receiving the test and, by extension, their immediate contacts (see right-hand column of Table 1).

Model parameters, listed in Table 2, represent the contextual factors to be examined (including plausible ranges for each), with the aim of identifying those factors that are most influential for the value of an Ag-RDT-led testing strategy relative to NAT-based testing. Our expert consultation highlighted that no standard guidance for whether or how Ag-RDTs should be used in conjunction with NAT existed at the time (e.g. whether NAT should be used to confirm an Ag-RDT negative result). Thus, we also defined and modelled three different options for the adjunctive use of NAT in an Ag-RDT-led algorithm: (i) no confirmation of Ag-RDT results, (ii) NAT confirmation of Ag-RDT negative results, or (iii) NAT confirmation of Ag-RDT positive results). Due to the lower sensitivity of Ag-RDT compared to NAT, confirmation of Ag-RDT negative results with NAT reduces the probability of false negatives, whereas confirmation of Ag-RDT positive results reduces the probability of false positives. The latter is especially important in settings with a low prevalence of COVID-19, where even small shortfalls in specificity can lead to substantial numbers of false positive diagnoses [32].

**Table 2:** Contextual parameters and their uncertainty ranges

Parameter	Value		References
	Hospital setting	Community setting	
*Epidemiology*			
Prevalence of current or recent SARS-CoV-2 infection (%) *	25	5	Assumption
**Proportion amongst those tested who are in acute phase**	0.5–1.00	0.5–1.00	Assumption
**Of those in acute phase, number of infectious days remaining (days)**	5–15	5–15	[16]
**Case fatality rate amongst hospitalised COVID-19 patients**	0.20–0.30	N/A	[7]
**Case fatality reduction amongst COVID-19 patients on dexamethasone (1—risk ratio)**	0.07–0.25	N/A	[7]
*NAT performance*			
**NAT sensitivity (for current or recent SARS-CoV-2)**	0.85–0.95	0.85–0.95	[17–23]
**NAT specificity**	0.99–1	0.99–1	[17–20]
**NAT availability (proportion able to access NAT test)**	0.1–1	0.1–1	Assumption
**Cost per NAT test ($)**	20–70	20–70	
**NAT turnaround time (days)**	1–10	5–15	[10], Expert consultation
**Confirm Ag-RDT negative results with NAT**	Y/N	Y/N	
Confirm Ag-RDT positive results with NAT **	Y/N	Y/N	
**Isolate and initiate treatment (if indicated) whilst awaiting NAT result**	Y	N	
*Ag-RDT performance (assumed fixed)*			
Ag-RDT sensitivity for current infection, relative to NAT (%, assumed only amongst acute cases)*	0.80	0.80	[14, 15]
Ag-RDT specificity, relative to NAT (%)*	0.98	0.98	[14, 15]
**Cost per Ag-RDT test ($)**	5	5	[14]
*Clinical judgement and management*			
**Sensitivity of clinical judgement in absence of NAT**	0.45–0.99	0.45–0.99	[24–27]
**Specificity of clinical judgement in absence of NAT**	0.20–0.70	0.20–0.50	[24–27]
**Proportion of hospitalised patients with a negative COVID-19 test result (true and false negatives) that are initiated onto dexamethasone**	0.05–0.15	N/A	Assumption
**Duration of isolation (days)**	10	10	[16]
**Duration of dexamethasone treatment (days)**	10	N/A	[7]
**Cost of isolation per day ($)**	50–350	N/A	[28–30]
**Cost of dexamethasone per day ($)**	0.13–3.5	N/A	[31]

Ranges define limits on uniform distributions, chosen to capture plausible parameter ranges that may apply across a variety of low- and middle-income settings. As described in the main text, the main analysis is a systematic uncertainty analysis, structured to identify which of these uniform distributions is most influential for model outcomes. *We performed sensitivity analyses on these fixed parameters, with results presented in the supporting information. We varied prevalence in the hospital setting between 10 and 30% and in the community setting between 1 and 10% (Additional file 7: Fig.S4). We varied Ag-RDT sensitivity and specificity between 75-95% and 98–100%, respectively, relative to NAT (Additional file 8: Fig.S5). **We exclude any parameter draws involving NAT confirmation of *both* Ag-RDT negative and Ag-RDT positive results.

For the hospital setting, we assumed that all patients are isolated while awaiting NAT results (whether in the NAT-based strategy or while awaiting NAT confirmation in an Ag-RDT-led strategy). In the supporting information we also present a sensitivity analysis of the alternative scenario where patients are not isolated while awaiting test results. By contrast, in the community setting we assumed that individuals are *not* isolated while awaiting any NAT result, as this policy was considered infeasible due to the comparatively low prevalence of COVID-19 in this population, and the unnecessary expense and disruption this would entail to most tested individuals and their families.

Although NAT specificity is near 100% for current or recent infection [17], not all NAT-positive cases are necessarily infectious, given the potential to detect unviable viral genetic material after the infection has resolved [17–19] and for severe symptoms to develop near the end of the infectious period [33]. By contrast, Ag-RDTs may detect only acute, but not recently cleared, infection [34, 35]. These distinctions have significance for the intended purpose of the test: where the purpose is to guide clinical decisions for treatment, knowing the aetiology of severe symptoms is important, regardless of viral antigenic load. On the other hand, where the purpose is early identification of infectious cases, detecting recently cleared infection can detract from the utility of a test. We captured these elements of both NATs and Ag-RDTs by distinguishing ‘acute’ from ‘recent’ infection and assuming that (i) only acute infection is infectious, (ii) NAT is able to detect both acute and recent infection with equal sensitivity and (iii) an Ag-RDT is able to detect only acute infection [34]. We accommodated wide uncertainty in the proportion of patients with acute infection in both the hospital and community settings; considering that viral load is highest just before symptom onset, and that the average COVID-19 patient is hospitalised 3 to 10 days after symptom onset [36], it is possible that a certain proportion of patients are no longer infectious by the time they are hospitalised. As discussed below, although these are useful simplifications for the purpose of the current analysis, these categorisations conceal potentially important complexities relating to temporal and between-individual variation in viral load, infectivity, and detectability by a given test. In the present analysis, we incorporated a parameter for the proportion of those with COVID-19, amongst the population being tested, that are still in the acute phase at the point of testing, allowing this parameter to occupy a wide range of values between 50% and 100%, acknowledging the existing uncertainty (See Table 2).

#### Quantifying relative value

In the hospital setting, we assumed that the test would guide decisions about whom to isolate and whom to treat with dexamethasone [37] and moreover that all patients (regardless of test result) would receive supportive care such as oxygen support. We assumed a baseline of no COVID-19-specific intervention (i.e. supportive care, but with no NAT or Ag-RDT testing strategy, nor treatment with dexamethasone). We assumed that hospitalised COVID-19 patients have a case fatality rate between 20 and 30% [7] without treatment, and that dexamethasone reduces this by 7–25% (Table 2), consistent with recent study results for corticosteroid treatment of COVID-19 [7]. We assume a 10-day treatment course of dexamethasone [7]. We thus denoted ‘deaths averted’ as the reduction in deaths that would be achieved by a given testing strategy, relative to no intervention.

Similarly, as a simple proxy for the impact of a test on transmission in both hospital and community settings, we first assumed a uniform distribution for the number of infectious days remaining per patient amongst patients presenting with acute infection (Table 2). We then recorded the number of patient days of acute infection that were not spent in isolation, whether because of missed diagnosis or (in the case of NAT) delayed diagnosis without isolation, while awaiting a test result. We denoted ‘infectious person-days averted’ as the reduction that would be achieved by a given testing strategy, relative to no-intervention baseline. For the community setting, we estimated the impact of a test only in terms of infectious person-days averted, as it is likely that most individuals receiving a test in a community setting suffer from mild COVID-19.

We also estimated the cost to the health system of the different interventions. For the hospital setting, we estimated the cost of testing, treatment and isolation. For the community setting, we estimated only the cost of testing.

Using the model illustrated in Fig. 1, we estimated the impact (deaths or infectious person-days averted) and cost of each testing strategy. We stratified Ag-RDT-led strategies by the adjunctive role of NAT in confirmation of a test result (i.e. whether to confirm Ag-RDT-negatives, Ag-RDT-positives, or not at all). For NAT-based strategies, we assumed that only a proportion of eligible individuals receive a NAT result (assuming a broad range of 10–100%), with the remainder managed through clinical judgement alone. For each use case, we sampled all parameters from the uncertainty ranges in Table 2 using Latin Hypercube Sampling. For each sampled set of parameters, we calculated both incremental costs and the incremental primary outcome (deaths averted or infectious person-days that were isolated) under an Ag-RDT-led strategy or a NAT-based strategy, relative to no intervention (that is, a scenario of no testing, nor clinical management of COVID-19). To quantify uncertainty, we calculated uncertainty intervals (UIs) as 2.5th and 97.5th percentiles over 10,000 samples and reported median values as point estimates.

To compare testing strategies, we first estimated the cost per death averted (in the hospital setting), and the cost per infectious person-day isolated (in both hospital and community settings) under NAT-based and Ag-RDT-led strategies. However, we did not aim to determine whether or not an Ag-RDT would be cost-effective, given the uncertainties surrounding appropriate willingness-to-pay thresholds for emergency outbreak response [38]. Instead, we compared the two strategies (Ag-RDT vs NAT) using a simple approach of plotting their relative impact against their relative cost for each sampled set of parameters (see Additional file 3: Fig.S1 for a schematic illustration of the approach). It is important to note that this approach is distinct from a conventional cost-effectiveness plane, as the axes are shown on a relative, rather than a nominal, scale. In the example of deaths, we denoted *A*_*RDT*_ as the deaths averted by Ag-RDT-led testing, relative to no intervention, and likewise for *A*_*NAT*_. Similarly, we calculated the incremental cost *C*_*RDT*_ of an Ag-RDT-led strategy relative to no intervention, and likewise for *C*_*NAT*_. We then plotted the relative impact (*A*_*RDT*_/*A*_*NAT*_) against the relative incremental cost (*C*_*RDT*_/*C*_*NAT*_).

We defined an Ag-RDT as being ‘favourable’ relative to NAT, wherever its use resulted simultaneously in more deaths averted than NAT (i.e. *A*_*RDT*_ > *A*_*NAT*_), and a lower incurred cost per death averted than NAT (i.e. *C*_*RDT*_/*A*_*RDT*_ < *C*_*NAT*_/*A*_*NAT*_). We defined an Ag-RDT as being ‘non-favourable’ otherwise. We performed corresponding calculations for the outcome of infectious person-days successfully isolated. Our focus in the following analysis is on identifying which circumstances would lead to an Ag-RDT being ‘favourable’ relative to NAT.

Where simulation outputs were equivocal on the favourability of Ag-RDTs, i.e. straddling favourable and non-favourable regions, we evaluated the correlation between each parameter and relevant model outputs using partial rank correlation coefficients (PRCC), to identify those parameters that were most influential on the proportion of a simulation falling in a favourable region. In brief, PRCC is an efficient approach to global sensitivity analysis that quantifies the strength of association between any given parameter and model output, while simultaneously taking account of variation in all other parameters (for more details on its implementation, see refs [39, 40]). In particular, where simulation outputs straddled the vertical dashed line shown in Additional file 3: Fig.S1, we evaluated correlations against the relative impact of Ag-RDT-led vs NAT-based testing strategies. Where simulations straddled the diagonal line in the upper-right quadrant, we evaluated correlations against the relative cost-per-unit impact (i.e. per death averted or per infectious person-day isolated). Overall, in this way, we sought to identify the contextual conditions under which an Ag-RDT-led strategy would, and would not, be favoured over NAT.

#### Additional sensitivity analysis

Finally, we analysed sensitivity to model assumptions not covered by the analyses above. As a focal model output for this sensitivity analysis, we chose the proportion of simulations that were favourable, under a given use case and a given scenario for the adjunctive use of Ag-RDT and NAT. First, while the main analysis adopted fixed values for sensitivity and specificity of an Ag-RDT, sensitivity analyses examined how the proportion favourable would vary if Ag-RDT sensitivity ranged from 75 to 95% and if Ag-RDT specificity ranged from 98 to 100%. Second, we examined how the proportion favourable would vary if prevalence of COVID-19 ranged from 10 to 30% in the hospital setting and 1–10% in the community setting. Finally, for simplicity in the main analysis of the community setting, we assumed perfect adherence to isolation after a positive test result and no self-isolation amongst those testing negative. We relaxed these assumptions in the sensitivity analysis and assumed that non-compliance with self-isolation reduced the infectious days averted by a proportion p, while those not required to isolate (i.e., those with false-negative test results, and those awaiting NAT confirmation of an initial Ag-RDT result in the community) nevertheless self-isolated to a degree that reduced transmission by a factor q. We examined how the proportion favourable varied as either p or q ranged from 0 to 50%.

#### Role of the funding source

This work was funded by the Foundation for Innovative New Diagnostics (FIND), through a grant from WHO. Authors JS and SS are employees of FIND. Otherwise, neither FIND nor WHO had no role in the study design, analysis, or interpretation.

## Results

As context to the primary analysis that follows, Additional file 4: Table S1 illustrates that both NAT-based and Ag-RDT-based algorithms were more costly, but led to better health outcomes, than a scenario of no intervention. For example, a NAT-based algorithm in a hospital setting would cost $150,000 (95% uncertainty intervals (UI) 38,000–490,000) per death averted within the patient population, while an Ag-RDT-led algorithm, involving NAT confirmation of Ag-RDT-negatives, would cost $140,000 (36,000–440,000). Likewise, in a community setting, a NAT-based algorithm was estimated to cost $84 (11–670) per infectious person-day isolated, versus $12 (8–23) for an Ag-RDT-only algorithm (without NAT confirmation).

### Hospital setting

**Fig. 2:**
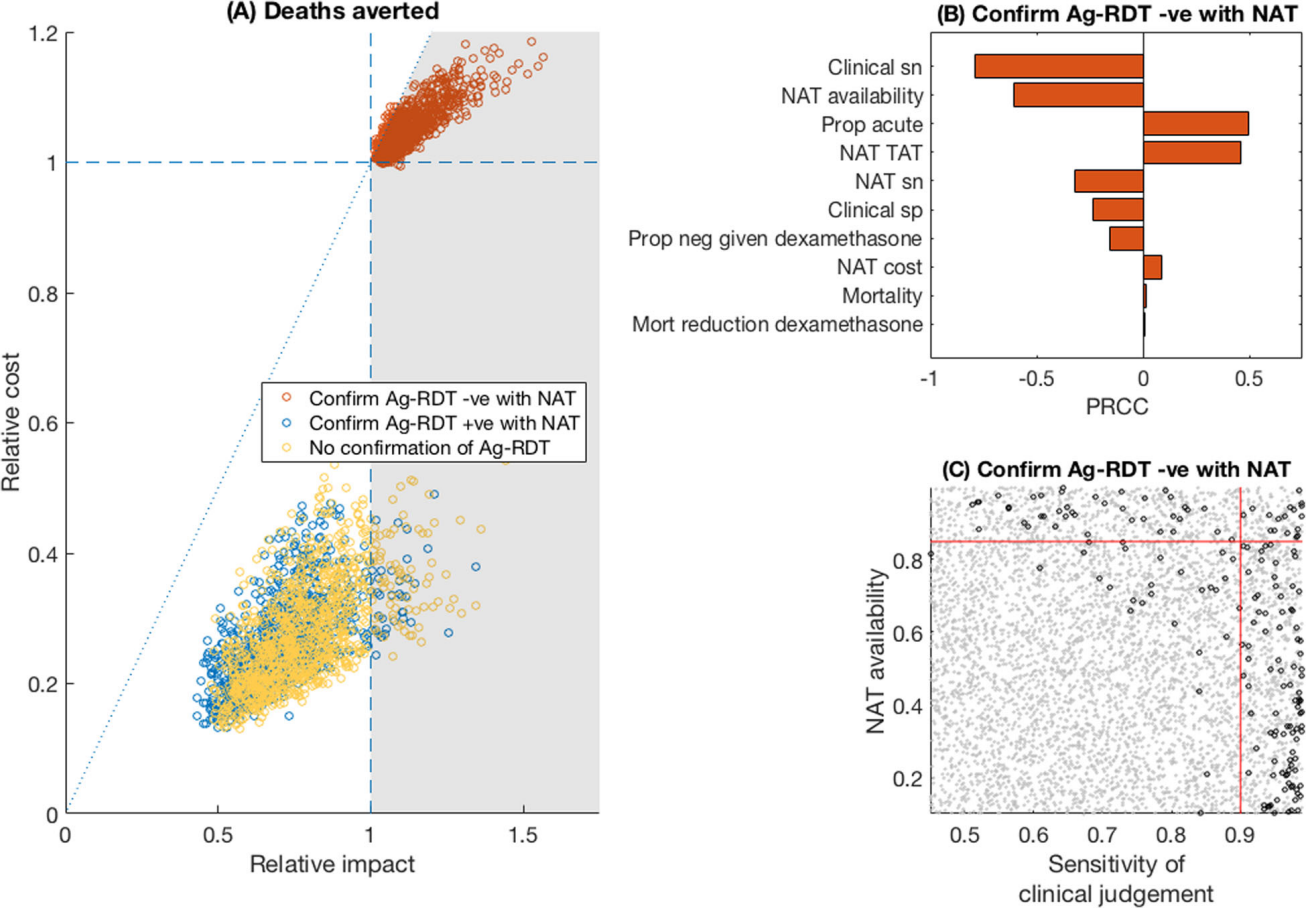
Relative value of Ag-RDT vs NAT testing, for averting deaths in a hospital setting. **a** Scatter plots for the relative impact of Ag-RDT vs NAT (horizontal axis) vs the relative cost of the two strategies (vertical axis). Each dot represents a single simulation with parameter values drawn from the ranges in Table 2. The grey-shaded area shows the region where an Ag-RDT-led strategy was ‘favourable’ over a NAT-only strategy, meaning that it averted more deaths, and at a lower cost per death averted (Additional file 3: Fig.S1). Colours of points indicate the adjunctive, confirmatory role of NAT in an Ag-RDT-led strategy (see in-figure legend). Of the red points, 96% fell in the favourable region. **b** Sensitivity analysis on the red points in **a**, to assess when these points fell above, or below, the diagonal dotted reference line. PRCC denotes ‘partial rank correlation coefficient’, against the cost per death averted. The longest bars indicate the most influential parameters; positive values indicate parameters that increased the favourability of the algorithm with increasingly positive values, and conversely for negative PRCCs. For example, when NAT was used to confirm negative results, the favourability of an Ag-RDT-led strategy was improved in settings having lower clinical sensitivity and a higher proportion of acute infection. **c** The joint role of the two most influential parameters in **b**. Grey and black points show parameter combinations where an Ag-RDT was favourable, and non-favourable, respectively, relative to NAT. Red lines show 90% sensitivity of clinical judgement (vertical line), and 85% NAT availability (horizontal line). In the lower left quadrant of these lines, an Ag-RDT was favourable over NAT in 99% of simulations. In these results, it is assumed that patients were placed in isolation (where indicated) while awaiting a NAT result: Additional file 5: Fig.S2 in the supporting information shows results in the alternative scenario where they were not isolated, pending NAT results

Figure 2 shows plots of relative incremental cost against relative impact in terms of deaths averted, comparing the Ag-RDT-led to the NAT-based strategy in a hospital setting, with an assumed 25% prevalence of acute or recent COVID-19 amongst those being tested. For deaths averted, when an Ag-RDT was used in conjunction with NAT to confirm Ag-RDT-negative results (red points), such a strategy had greater impact, and at lower cost per death averted, than a NAT-based strategy (‘favourable region’) in 96% of all simulations. By contrast, Ag-RDT-led strategies that involved either no NAT confirmation, or only confirmation of RDT-positive cases (respectively yellow and blue points), resulted in too many missed cases to exceed the impact of NAT-based strategies in more than 92 and 96% of simulations, respectively. For settings in which NAT was used to confirm Ag-RDT-negative results, Fig. 2b illustrates the relationship of each model parameter to the proportion of parameter samples that resulted in a ‘favourable’ simulation. In particular, the availability of NAT, sensitivity of clinical judgement amongst those unable to access NAT, and proportion of cases tested during the acute phase were highly influential. Figure 2c shows the most influential parameters (NAT availability and clinical judgement) in greater detail, with points in grey showing where an Ag-RDT was favourable. Broadly, the figure illustrates that an Ag-RDT would be favourable in settings of low NAT availability and low sensitivity of clinical judgement: in indicative terms, as long as sensitivity of clinical judgement was < 90%, and NAT was available to < 85% of patients, 99% of simulations were favourable.

**Fig. 3:**
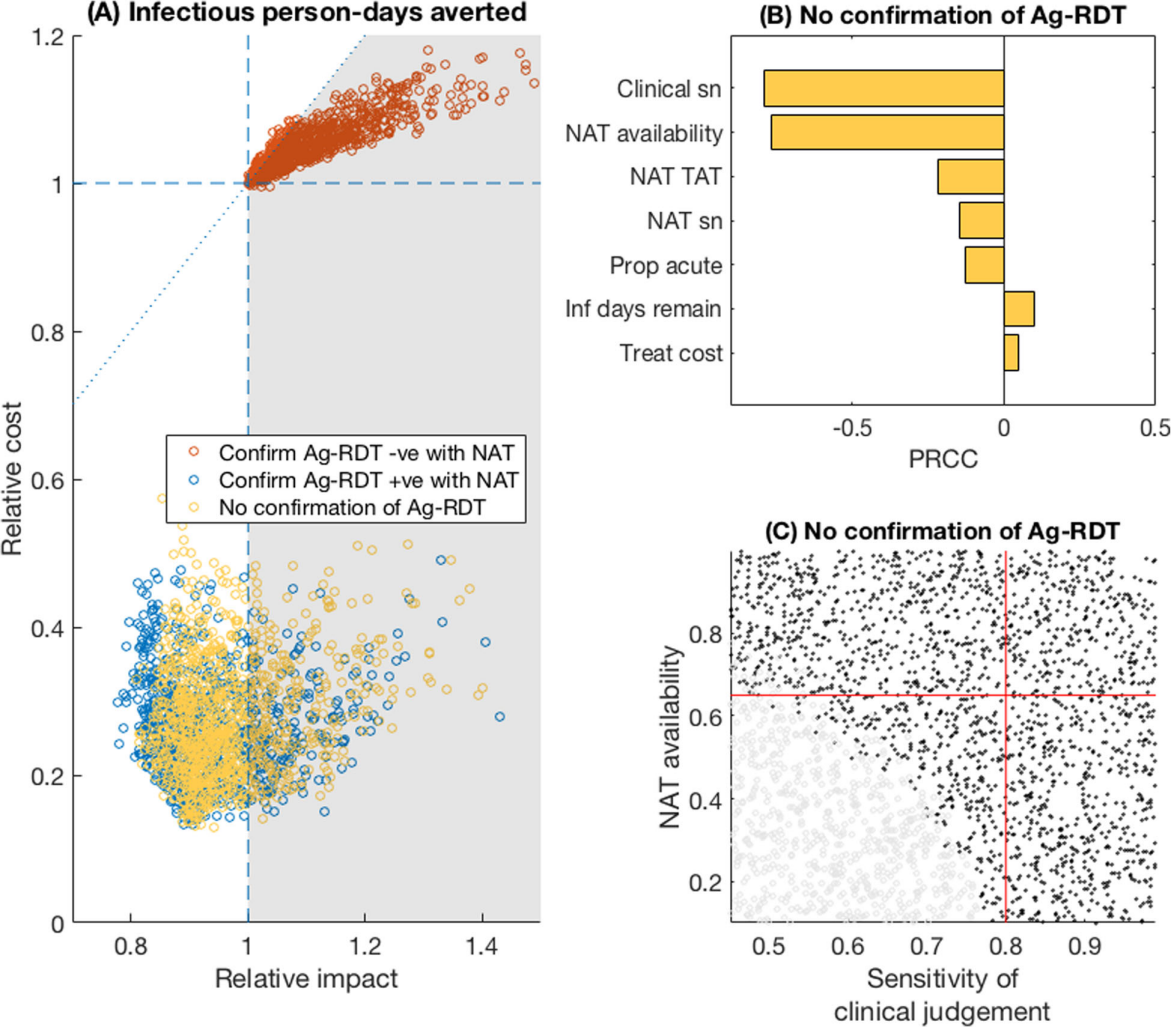
Relative value of Ag-RDT vs NAT testing, for averting infections in a hospital setting. **a** Scatter plots for the relative impact of Ag-RDT vs NAT (horizontal axis) vs the relative cost of the two strategies (vertical axis). Of the yellow points (no NAT confirmation of Ag-RDT results), 27% fell in the favourable region shaded in grey. Details as in Fig. 2a and Additional file 3: Fig.S1. **b** Sensitivity analysis for model parameters on the yellow points in **a**. The interpretation of PRCC is explained in further detail in the caption of Fig. 2. **c** concentrates on the two most influential parameters in this case, NAT availability and sensitivity of clinical judgement. As in Fig. 2c, grey and black points show parameter regimes where an Ag-RDT was, respectively, favourable and unfavourable, relative to NAT. Red lines show 80% sensitivity of clinical judgement (vertical line) and 65% NAT availability (horizontal line). In the lower left quadrant of these lines, an Ag-RDT was favourable over NAT in 66% of simulations. In these results, it was assumed that patients were placed in isolation while awaiting a NAT result: Additional file 6: Fig.S3 in the supporting information shows results in the alternative scenario where they were not isolated

Figure 3 shows similar results for the outcome of infectious person-days isolated in a hospital setting. Importantly, Fig. 3a illustrates the potential for the sole use of Ag-RDT (without NAT confirmation) to offer higher impact, at lower cost, than a NAT-based scenario (yellow points, 27% of which were in the favourable region). Figure 3c shows a bivariate sensitivity analysis of the two most influential model parameters, demonstrating that an Ag-RDT-only strategy was likely to be favourable in terms of averting infection as long as the sensitivity of clinical judgement in the absence of NAT was < 80% and the availability of NAT was < 65%. Under these conditions, the proportion of simulations that were favourable was 66%.

These results assume that all patients were placed in isolation while awaiting NAT results. Additional file 5: Fig.S2 and Additional file 6: Fig.S3 show corresponding results when assuming no isolation pending NAT results, illustrating that the results are essentially unchanged for deaths averted (Additional file 5: Fig.S2). For infectious patient days isolated, a practice of isolating patients pending NAT results mitigated the drawbacks of multi-day NAT turnaround times: a decision not to isolate pending results therefore reduced the impact of a NAT-based strategy, thus making an Ag-RDT-only strategy more favourable in comparison. Additional file 6: Fig.S3 illustrates that, in such a scenario, 93% of simulations placed the Ag-RDT strategy in the favourable region.

### Community setting

**Fig. 4:**
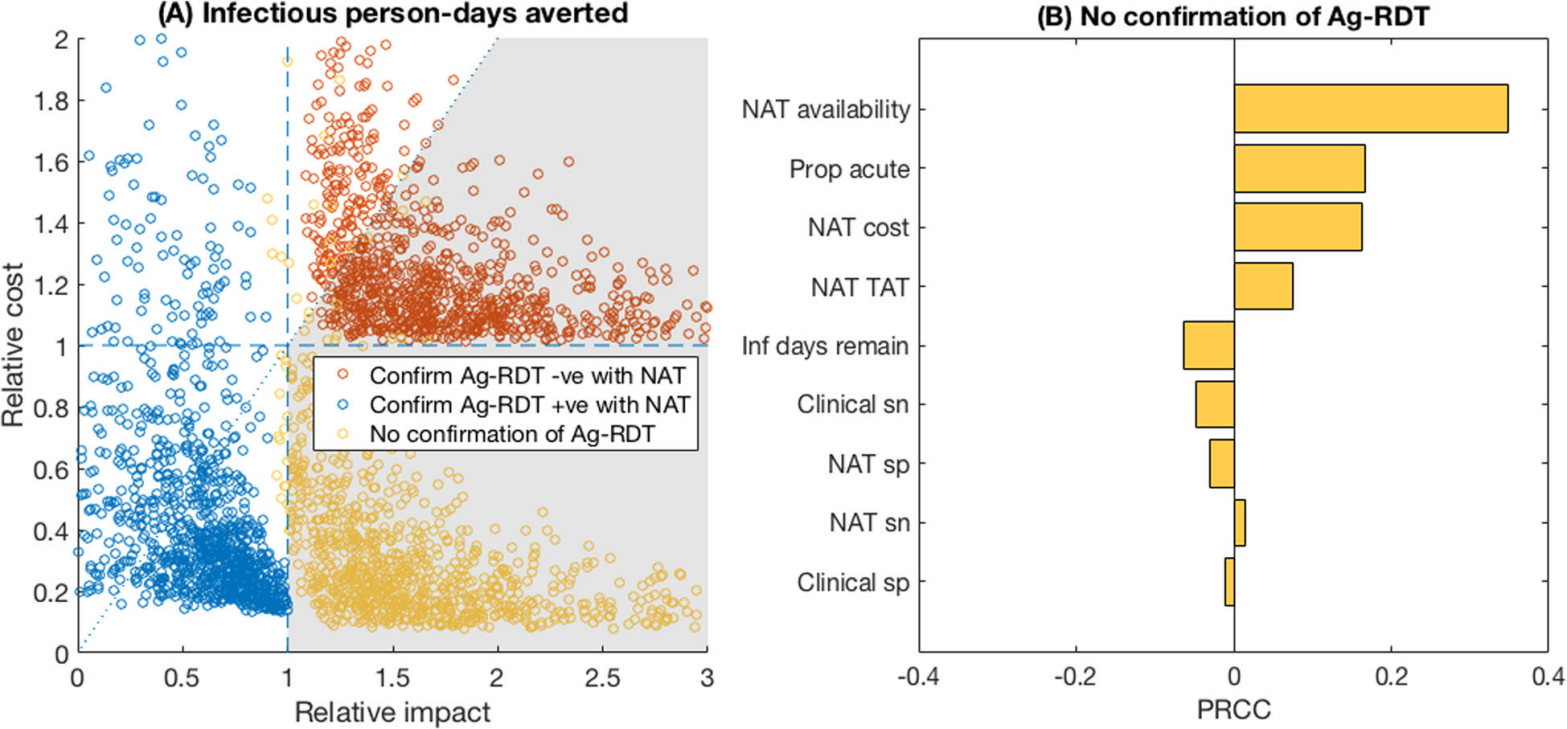
Relative value of Ag-RDT vs NAT testing in a community setting. We assumed that in a community setting, the focus is on averting infection, and that any severe cases of respiratory disease are more likely to present in hospital settings (Fig. 3). Hence, in this setting, we focused on infectious person-days averted; we also assumed that individuals awaiting NAT results were not isolated during this time, owing to the infeasibility of doing so in this setting. **a** Scatter plot of the relative impact of Ag-RDT vs NAT (horizontal axis) vs the relative cost of Ag-RDT vs NAT (vertical axis). Dashed reference lines are as explained in Fig. 2 and in Additional file 3: Fig.S1. Of the yellow points (no NAT confirmation of Ag-RDT results), 98% fell in the favourable region shaded in grey; of the red points (confirm Ag-RDT negatives with a NAT), 80% fell in the favourable region. **b** Subgroup sensitivity analysis of the yellow points in **a**. Interpretation of PRCCs are as explained in Fig. 2 caption. Because the vast majority (98%) of simulations show Ag-RDT was favourable to NAT in this scenario, we did not conduct additional bivariate sensitivity analyses as for Figs. 2c and 3c

Finally, Fig. 4 shows results for the community setting scenario. Key assumptions, compared to the hospital scenario, include the sole priority is to avert infection, because mortality risk in the individuals being evaluated is low; lower prevalence of SARS-CoV-2 amongst those being tested (5%); and we assumed individuals are not placed in isolation while awaiting NAT results, because of the infeasibility of doing so. Similarly to Fig. 3, confirming Ag-RDT-negative cases with NAT was highly likely to avert more potential transmission than NAT alone, and at lower cost per infectious day averted (red points, favourable in 80% of simulations). It was also possible for the sole use of Ag-RDT to be more impactful than NAT while costing less (yellow points, favourable in 98% of simulations). Figure 4b illustrates the key drivers that increased the relative impact of Ag-RDT-only vs NAT-based strategies. These were a higher proportion of individuals that were still in their acute (infectious) phase while being tested, a higher availability of NAT, a higher cost per NAT test, and a longer NAT turnaround time.

### Additional sensitivity analysis

Additional file 7: Fig.S4, Additional file 8: Fig.S5 and Additional file 9: Fig.S6 show additional sensitivity analyses for both hospital and community settings, under alternative model assumptions. For example, the proportion of simulations being favourable for Ag-RDT remained stable with respect to alternative assumptions for the prevalence of COVID in the population being tested and under different algorithms, in both hospital and community settings (Additional file 7: Fig.S4). Increasing Ag-RDT sensitivity increased the favourability of all three Ag-RDT algorithms in the hospital setting (Additional file 8: Fig.S5A, B.), but had only modest effect on the community setting (Additional file 8: Fig.S5C). Increasing Ag-RDT specificity had similarly modest impact on an algorithm’s favourability in all settings (Additional file 8: Fig.S5D-F). Additional file 9: Fig.S6 shows analysis under alternative assumptions for self-isolating behaviour in the community setting. Diminishing compliance with requirements to self-isolate tended to reduce the favourability of an Ag-RDT-only strategy (i.e. with no NAT confirmation), but had the opposite effect on the favourability of a strategy to confirm Ag-RDT-positives. In both cases, the proportion favourable varied by less than 10 percentage points over the parameter range examined here. Similar sensitivity was observed for model assumptions relating to voluntary self-isolation, when not required to do so (Additional file 9: Fig.S6B).

## Discussion

The emergence of antigen-detection rapid diagnostic tests for SARS-CoV-2 has raised important questions about trade-offs between accessibility and performance; to inform country-level decisions about the use of these tests, there is a need for more evidence on how to navigate such trade-offs. Recent models and commentaries have highlighted the potential utility of high-frequency, low-sensitivity testing of asymptomatic individuals [41], and the current analysis demonstrates that under certain circumstances, a less-sensitive but more-accessible test may be preferable for diagnosis of symptomatic COVID-19 as well. Rather than aiming to specify parameter values with precision, our approach instead embraces parameter uncertainty, by modelling a broad range of scenarios or contextual factors. This approach partly reflects the uncertainty in model parameters, but also their anticipated variability across different country settings and as local epidemics change over time. By structuring our approach in this fashion, we sought to identify the contextual factors that are most important in deciding the value of an Ag-RDT.

Our results suggest that the value of an Ag-RDT-led strategy is strongly supported for evaluating symptomatic individuals in community settings, being highly likely to be simultaneously less costly and more impactful than relying on NAT and clinical judgement (Fig. 4). In hospital settings, the favourability of Ag-RDT may be subject to certain qualifications. For example, in averting deaths, an Ag-RDT, supported by NAT to confirm Ag-RDT-negative results, is likely to be favourable (averting more deaths, at less cost per death averted) to NAT and clinical judgement alone, in settings where NAT is available for less than 85% of patients and sensitivity of clinical judgement (in the absence of NAT) is less than 90% (Fig. 2). However, although confirmation of a negative Ag-RDT result with NAT averts more deaths for a given cost than NAT only, this algorithm is still more costly than a NAT-only algorithm and may therefore raise challenges of affordability in settings with limited resources.

We note that in the community setting in particular, any reliance on NAT-based testing would face substantial challenges in practice. For example, in most settings, it is unlikely that individuals would be adequately isolated while awaiting NAT results, given the large number of unnecessary isolations, and associated burden on patients and families, that such a strategy would incur. Moreover, it will also typically be infeasible to offer timely NAT to all individuals with potential COVID-19 symptoms, given the attendant financial, human resource and supply constraints. In this setting, our analysis shows how an affordable, rapid test, even one with lower performance than NAT, can achieve greater impact overall, and at lower cost, than a strategy that relies on NAT instead.

Notably in both hospital and community scenarios, the key determining factors for the value of an Ag-RDT (namely, the availability of NAT, sensitivity of clinical judgement, and proportion of cases tested during the acute phase) all relate to the ability of the existing system to detect cases of SARS-CoV-2. These findings highlight the potential value of implementation studies to gather data on these factors when making programmatic decisions for the introduction and implementation of new Ag-RDTs in any given setting. Overall, this work serves broadly to illustrate an analytical framework that could be readily adjusted to local realities in different settings. A simple, user-friendly web-based tool is available, to perform the simulations shown here, but also to allow these simulations to be extended to alternative, user-specified parameter ranges [13].

Certain limitations of scope bear mention. Our focus in this work is on identifying the circumstances in which an Ag-RDT might be most valuable, given a pre-specified performance profile. Recent guidance published by WHO addresses target product profiles for Ag-RDTs: that is, how a test should best be optimised in terms of accuracy, cost and ease-of-use, for specified use cases [11]. For simplicity, our approach treats transmission-related impact of testing as being directly proportional to the number of days for which testing results in isolation of an infectious person, without considering variation between individuals or over time in the degree of infectivity or the strictness of isolation. Similarly, our assessment of mortality outcomes does not account for the potential of a test to indirectly reduce incidence and mortality by interrupting transmission. Further work using dynamic models of SARS-CoV-2 transmission would be valuable in addressing this gap. In addition, while our results are based on a broad sensitivity analysis, it should be noted that these same results may depend on the range of parameters that we have assumed, and indeed these ranges may vary across different settings. Our user-friendly tool allows users to adapt some of these ranges to specific settings. Amongst other limitations, we have adopted several simplifications, perhaps most importantly assuming a dichotomy between ‘acute’ and ‘recent’ infection and the detectability of each by NAT or Ag-RDT. This assumption ignores potentially important complexities, including how infectivity varies over the clinical course; the stage in the clinical course at which individuals are likely to be tested; and the implications of changing viral/antigen/RNA load over the clinical course, for the ability of a given test to detect infection [18, 42–45]. Previous modelling studies have incorporated some combinations of these factors [3, 41], but longitudinal data on all of these factors will be critical in refining these and other modelling approaches, to account fully for their potential interactions.

## Conclusions

Given the immediate importance of virological testing for the control of SARS-CoV-2, it is important for decisions about testing strategy to be guided by the available evidence. Our results show how, in certain clinical conditions, the use of Ag-RDTs could achieve equal or greater impact, and at lower cost, than relying on NAT alone. While the accuracy of diagnostic tools is important, other considerations are also critical: as control efforts increasingly shift from blanket lockdowns towards intensive testing and early identification, the speed, affordability, and ease-of-use of diagnostic tools are likely to play an increasingly key role in the response to SARS-CoV-2. Our findings illustrate where such rapid and affordable tests are likely to improve outcomes, in a more cost-efficient way than reliance on NAT and clinical judgement alone.

## Supplementary Information

**Additional file 1: Text S1.** Expert consultation.

**Additional file 2: Text S2.** Model equations.

**Additional file 3: Figure S1.** Schematic illustration for visualising the value of an Ag-RDT-led strategy, relative to a scenario involving NAT and clinical judgement. Although the figure involves deaths averted, the same structure applies for averting infectious person-days. For a given set of parameters drawn from the parameter ranges shown in Table 2, we simulated the cost and impact of a given Ag-RDT-led strategy, and of a NAT-based testing strategy, both relative to a no-intervention scenario. This outcome was then represented in the figure by plotting the relative deaths averted by Ag-RDT vs NAT (horizontal axis) against the relative cost of the two strategies (vertical axis). Thus, for example, in the lower right quadrant, an Ag-RDT-led strategy would cost less, but have more impact, than NAT. The diagonal dashed line shows an important threshold: for points below this line, an Ag-RDT-led strategy would cost less per death averted than NAT, and vice versa. Overall, therefore, the shaded area shows the region in which an Ag-RDT would simultaneously cost less per death averted, and avert more deaths overall, than NAT. We denote this area as the ‘favourable region’ for an Ag-RDT, and elsewhere as ‘non-favourable’: in our current analysis we aim to identify the circumstances under which an Ag-RDT, of a given performance and cost, would occupy this region.

**Additional file 4: Table S1.** Summary of cost per death or infectious person-day averted of results presented in the main text.

**Additional file 5: Figure S2.** Relative value of Ag-RDT-led vs NAT-based testing, for averting deaths in a hospital setting. The figure shows the same results as those presented in Fig. 2 in the main text, but here assuming that all patients awaiting a NAT result (whether as part of a NAT-based strategy or for confirmation of Ag-RDT results) were *not* isolated during this time. Results illustrate qualitatively similar findings to those shown in the main text. In panel (A), in the scenario where Ag-RDT-negative results were confirmed using NAT (red points), 57% of simulations placed the Ag-RDT-led strategy in the favourable region, below the diagonal dashed line. Panels (B, C) show additional sensitivity analyses for these points in particular, as described in Fig. 2. In (C), red lines show 75% NAT availability (vertical line), and 90% sensitivity of clinical judgement (horizontal line). In the lower left quadrant of these lines, an Ag-RDT was favourable over NAT in 85% of simulations.

**Additional file 6: Figure S3.** Relative value of Ag-RDT-led vs NAT-based testing, for averting infections in a hospital setting. The figure shows the same results as those presented in Fig. 3 in the main text, but here assuming that all patients awaiting a NAT result (whether as part of a NAT-based strategy or for confirmation of Ag-RDT results) were not isolated during this time. In panel (A), in the scenario where there was no NAT confirmation of Ag-RDT results (yellow points), 93% of simulations placed the Ag-RDT-led strategy in the favourable region, to the right of the vertical, dashed line. Panels (B, C) show additional sensitivity analyses for these points in particular, as described in Fig. 3. In (C), red lines show a NAT turnaround time of 3 days (vertical line), and a 30% NAT availability (horizontal line). In the upper right quadrant of these lines, an Ag-RDT was favourable over NAT in 69% of simulations.

**Additional file 7: Figure S4.** Sensitivity analysis to varying prevalence of COVID-19 amongst those being tested. As a focal model output, all figures show the proportion of simulations in which an Ag-RDT was favourable, with different algorithms labelled by the different line colours. Panels A and B show the impact of varying prevalence on deaths and infectious days averted, respectively, in a hospital setting. Panel C shows the impact on infectious days averted in a community setting. Similar to the analysis presented in the main text, we assumed that all individuals were isolated whilst waiting for a NAT result in the hospital setting and that no one isolated whilst awaiting a test result in the community setting. Results illustrate that the proportion favourable remained stable to these alternative assumptions for prevalence.

**Additional file 8: Figure S5.** Sensitivity analysis to varying Ag-RDT sensitivity and specificity. As a focal model output, all figures show the proportion of simulations in which an Ag-RDT was favourable, with different algorithms labelled by the different line colours. Panels A-C show the sensitivity of Ag-RDT being varied between 75 and 95% across the hospital and community settings, assuming specificity remained fixed at 98%. Panels D-F show the specificity of Ag-RDT being varied between 98 and 100%, assuming sensitivity remained fixed at 80%. Similar to the analysis presented in the main text, we assumed that all individuals were isolated whilst waiting for a NAT result in the hospital setting and that no one isolated whilst awaiting a test result in the community setting. Results illustrate that, in a community setting, increasing Ag-RDT sensitivity increased the favourability of the “Ag-RDT only” and “confirm Ag-RDT negative” strategies (panel C). For example, the favourability of an algorithm that confirms an Ag-RDT negative result increased from 79% to 83% when sensitivity increased from 75% to 95%. Increasing sensitivity had little impact on the “confirm Ag-RDT positive” strategy; since the only costs incurred under a community setting was the cost of a test, a NAT-only strategy was often cheaper and averted more infectious days than the “confirm Ag-RDT positive” strategy (the cost of testing with an Ag-RDT and confirming a positive result with a NAT test makes it costly, and by re-testing a positive result with a NAT, the sensitivity of the algorithm was lower due to the imperfect sensitivity of NAT). Similar to the hospital setting, specificity had little impact on an algorithm’s favourability (panel F).

**Additional file 9: Figure S6.** Sensitivity analysis to patient behaviour in relation to self-isolation, in the community setting. As a focal model output, all figures show the proportion of simulations in which an Ag-RDT was favourable, with different algorithms labelled by the different line colours. Panel A shows the impact of compliance amongst those required to self-isolate after a positive final test result. Panel B shows the impact of test-negative individuals voluntarily self-isolating. This sensitivity analysis was restricted to the community setting as it is likely that hospitals will enforce compliance to isolation guidelines. Results illustrate that increasing the proportion of compliance to isolation recommendations increased the favourability of both “Ag-RDT-only” and “confirm Ag-RDT negative” strategies, from 86% and 68% of simulations being favourable with 50% compliance to 98% and 80% with 100% compliance, respectively. The benefit of an Ag-RDT test in rapidly detecting COVID cases, and hence averting onward transmission, is reduced if these individuals did not isolate. However, the opposite was seen with the “confirm Ag-RDT positive” strategy, with the favourability of the algorithm decreasing from 8% to 0% if compliance doubled from 50% to 100%. Generally, this strategy detected fewer COVID cases than a NAT-based strategy, due to the reduction in overall sensitivity caused by inclusion of NAT confirmation; thus, increasing the proportion of individuals that did comply had a greater effect on a NAT-based strategy than the Ag-RDT strategy, hence increasing the latter’s favourability. Similar results were seen for voluntary self-isolation (where false negatives voluntarily self-isolate).

## Data Availability

All data used in this study is available in the manuscript and supporting information.

## Abbreviations

NATs: Nucleic acid tests
Ag-RDTs: Antigen-detection rapid diagnostic tests
WHO: World Health Organization
FDA: The United States Food and Drug Administration
UIs: Uncertainty intervals
PRCC: Partial rank correlation coefficient
FIND: Foundation for Innovative New Diagnostics
