# Supplementary material for: Quantifying the potential value of antigen-detection rapid diagnostic tests for COVID-19: a modelling analysis
Source: BMC Med. 2021 Mar 9;19:75. doi: 10.1186/s12916-021-01948-z (PMC7939929; doi:10.1186/s12916-021-01948-z)
Supplement: Supplementary file 1 — Additional file 1: Text S1. Expert consultation. [file 12916_2021_1948_MOESM1_ESM.docx]

**Additional file 1: Text S1**

**Expert consultation**

To identify appropriate use cases for implementation of antigen-detection RDTs for SARS-CoV-2, we conducted a series of four expert consultations. For each consultation, we identified an in-country expert with knowledge of the landscape of diagnostic testing for infectious diseases and the country’s response to the COVID-19 pandemic. We consulted experts in Brazil, India, Nigeria, and South Africa (experts listed in Table A, below) – countries with large populations, a breadth of income levels, broad geographic representation, and COVID-19 epidemics of substantial magnitude. Consultations were performed between July 2 and 7, 2020. Each consultation was structured as follows:

First, we provided background on the potential characteristics of an Ag-RDT: results within 30 minutes, more feasible to perform at point of care, performed on oropharyngeal/nasal/salivary specimens, and less sensitive than nucleic acid amplification tests. Second, we described the primary research objective: to estimate the benefits and harms of implementing Ag-RDTs in specific use cases. Third, we provided examples of paradigmatic use cases, in terms of eligible population and intended use of the Ag-RDT. Fourth, we asked in-country experts to identify at least two important use cases for their country’s setting, asking that they define: (a) the eligible population; (b) the intended use/incremental benefit of Ag-RDT; (c) the status quo – how members of the population would be managed in the absence of an Ag-RDT; (d) how the status quo would change in the presence of an Ag-RDT (for both people testing positive and negative on Ag-RDT); (e) the most important outcomes/measures of value; and (f) the most important tradeoffs to consider.

Each consultation lasted between 60 and 90 minutes, resulting in the following use cases being identified:

- Community-based testing of symptomatics, in decentralised clinics or in dedicated testing facilities in containment zones (India and South Africa)
- Testing of symptomatic individuals in health facilities and amongst those being admitted to hospital (India and South Africa)
- Testing of asymptomatic employees contacting high-risk populations such as long-term care facilities (South Africa)
- Testing of symptomatic outpatients (Brazil and Nigeria)
- Testing of asymptomatic employees in high-risk occupations such as healthcare workers (Brazil and Nigeria)

For the purpose of this analysis, we focused on the first two use cases listed above. However, the approach described in our study can also straightforwardly be extended to the other use cases listed here.

| **Name of expert** | **Country** | **Affiliation** |
| --- | --- | --- |
| Kiran Rade | India | Indian Council of Medical Research |
| Dhamari Naidoo | Nigeria | WHO Country Office |
| Francois Venter | South Africa | WITS Reproductive Health and HIV Institute |
| Amilcar Tanuri | Brazil | Federal University of Rio de Janeiro |

**Table A. List of country experts consulted to identify appropriate use cases, for an Ag-RDT**
