# Supplementary material for: Quantifying the potential value of antigen-detection rapid diagnostic tests for COVID-19: a modelling analysis
Source: BMC Med. 2021 Mar 9;19:75. doi: 10.1186/s12916-021-01948-z (PMC7939929; doi:10.1186/s12916-021-01948-z)
Supplement: Supplementary file 2 — Additional file 2: Text S2. Model equations. [file 12916_2021_1948_MOESM2_ESM.docx]

**Additional file 2: Text S2**

**Model equations**

| Parameter | Notation |
| --- | --- |
| *Epidemiology* | |
| Prevalence of current or recent SARS-CoV-2 infection | $P$ |
| Proportion amongst those tested who are in acute phase | $p_{inf}$ |
| Of those in acute phase, number of infectious days remaining | $D_{inf}$ |
| Case fatality rate amongst hospitalised COVID-19 patients | $M$ |
| Case fatality reduction amongst COVID-19 patients on dexamethasone (1 – risk ratio) | $M_{red}$ |
| *NAT performance* | |
| NAT sensitivity (for current or recent SARS-CoV-2) | $N_{sn}$ |
| NAT specificity | $N_{sp}$ |
| NAT availability (proportion able to access NAT test) | $p_{NAT}$ |
| Cost per NAT test | $C_{NAT}$ |
| NAT turnaround time | $D_{NAT}$ |
| Isolate and initiate treatment (if indicated) whilst awaiting NAT result* | $I_{isol}$ |
| *Ag-RDT performance* | |
| Ag-RDT sensitivity for current infection, relative to NAT | $R_{inf\_sn}$ |
| Ag-RDT sensitivity for recent infection, relative to NAT | $R_{non\_inf\_sn}$ |
| Ag-RDT specificity, relative to NAT | $R_{sp}$ |
| Cost per Ag-RDT test | $C_{RDT}$ |
| Confirmation of an Ag-RDT negative result with NAT* | $I_{neg}$ |
| Confirmation of an Ag-RDT positive result with NAT* | $I_{pos}$ |
| *Clinical judgement and management* | |
| Sensitivity of clinical judgement in absence of NAT | $J_{sn}$ |
| Specificity of clinical judgement in absence of NAT | $J_{sp}$ |
| Proportion of hospitalised patients with a negative COVID-19 test result (true and false negatives) that are initiated onto dexamethasone | $p_{treat}$ |
| Duration of isolation | $D_{isol}$ |
| Duration of treatment | $D_{treat}$ |
| Cost of isolation per day | $C_{isol}$ |
| Cost of treatment per day | $C_{treat}$ |

**Table B. Parameter notation.** See table 2 in the main text for parameter values. *These variables are indicator variables (Yes = 1; No = 0); in other words, these variables switch components of the equations discussed below on and off depending on the algorithm selected.

For each algorithm, we calculated the costs, deaths averted, and infectious person-days averted. First, we calculated the probability of a positive diagnosis (true and false positives) and probability of a negative diagnosis (true and false negatives) for each algorithm. These probabilities were then multiplied by various parameters in order to calculate the various outputs.

**Probability of a positive diagnosis**

Below, are the probabilities of a positive diagnosis ($D_{p\_NAT}$ and $D_{p\_RDT}$) for each algorithm:

***NAT-based strategy***

$$\boldsymbol{D}_{\boldsymbol{p\_NAT}}=$$

$\left[ P\times p_{NAT}\times N_{sn} \right]+$ *1, True positive (NAT)*

$[P\times{(1-p}_{NAT})\times J_{sn}]+$ *2, True positive (clinical judgement)*

$[(1-P)\times p_{NAT}\times{(1-N}_{sp})]+$ *3, False positive (NAT)*

$[(1-P)\times{(1-p}_{NAT})\times{(1-J}_{sp})]$ *4, False positive (clinical judgement)*

Where $P$ is the prevalence of current or recent SARS-CoV-2 infection; $p_{NAT}$ is the proportion able to access a NAT test; $N_{sn}$ and $N_{sp}$is NAT sensitivity and specificity, respectively; $J_{sn}$and $J_{sp}$ is the sensitivity and specificity of clinical judgement in the absence of NAT, respectively.

***Ag-RDT-led strategy***

There are three different Ag-RDT led strategies: (1) Ag-RDT only strategy, (2) Confirmation of an Ag-RDT negative result with a NAT test, (3) Confirmation of an Ag-RDT positive result with a NAT test. The overall probability of receiving a positive test result as a result of an Ag-RDT-led algorithm can be calculated by the following framework, with different components of the framework being zeroed by indicator variables (I_neg_ and I_pos_) depending on which algorithm is selected:

*Framework:*

$$\boldsymbol{D}_{\boldsymbol{p\_RDT}}={[D}_{p\_RDT\_only}\times{(1-I}_{neg}-I_{pos})]+[D_{p\_RDT\_neg}\times I_{neg}]+{[D}_{p\_RDT\_pos}\times I_{pos}]$$

The individual probabilities ($D_{p\_RDT\_only}$, $D_{p\_RDT\_neg}$ and $D_{p\_RDT\_pos}$) can be calculated by the following equations:

*Ag-RDT only:* ${(D}_{p\_RDT\_only})$

$$\boldsymbol{D}_{\boldsymbol{p\_RDT\_only}}=$$

$\left[ P\times p_{inf}\times R_{inf\_sn} \right]+$ *1, True positive, infectious*

$[P\times{(1-p}_{inf})\times R_{non\_inf\_sn}]+$ *2, True positive, non- infectious*

$[(1-P)\times{(1-R}_{sp})]$ *3, False positive*

Where $P$ is the prevalence of current or recent SARS-CoV-2 infection; $p_{inf}$ is the proportion amongst those tested who are in acute phase; $R_{inf\_sn}$, $R_{non\_inf\_sn}$ and $R_{sp}$is the Ag-RDT sensitivity for current infection, Ag-RDT sensitivity for recent infection, and Ag-RDT specificity, respectively, all relative to NAT.

*Confirmation of an Ag-RDT negative with a NAT:* $(D_{p\_RDT\_neg})$

$$\boldsymbol{D}_{\boldsymbol{p\_RDT\_neg}}=$$

$[P\times p_{inf}\times R_{inf\_sn}]+$ *1, True positive (Ag-RDT), infectious*

$[P\times{(1-p}_{inf})\times R_{non\_inf\_sn}]+$ *2, True positive (Ag-RDT), non-infectious* $[P\times p_{inf}\times(1-R_{inf\_sn})\times p_{NAT}\times N_{sn}]+$ *3, True positive (NAT), infectious*

$[P\times{(1-p}_{inf})\times{(1-R}_{non\_inf\_sn})\times p_{NAT}\times N_{sn}]+$ *4, True positive (NAT), non-infectious*

$[P\times p_{inf}\times(1-R_{inf\_sn})\times{(1-p}_{NAT})\times J_{sn}]+$ *5, True positive (clinical judgement), infectious*

$[P\times{(1-p}_{inf})\times{(1-R}_{non\_inf\_sn})\times{(1-p}_{NAT})\times J_{sn}]+$ *6, True positive (C.J.), non-infectious*

$[(1-P)\times{(1-R}_{\mathrm{sp}})]+$ *7, False positive (Ag-RDT)*

$[(1-P)\times R_{\mathrm{sp}}\times p_{NAT}\times{(1-N}_{sp})]+$ *8, False positive (NAT)*

$[(1-P)\times R_{\mathrm{sp}}\times{(1-p}_{NAT})\times{(1-J}_{sp})]$ *9, False positive (C.J.)*

Where $P$ is the prevalence of current or recent SARS-CoV-2 infection; $p_{inf}$ is the proportion amongst those tested who are in acute phase; $R_{inf\_sn}$, $R_{non\_inf\_sn}$ and $R_{sp}$is the Ag-RDT sensitivity for current infection, Ag-RDT sensitivity for recent infection, and Ag-RDT specificity, respectively, all relative to NAT; $p_{NAT}$ is the proportion able to access a NAT test; $N_{sn}$ and $N_{sp}$is NAT sensitivity and specificity, respectively; $J_{sn}$and $J_{sp}$ is the sensitivity and specificity of clinical judgement in the absence of NAT, respectively.

*Confirmation of an Ag-RDT positive with a NAT:* $(D_{p\_RDT\_pos})$

$$\boldsymbol{D}_{\boldsymbol{p\_RDT\_pos}}\boldsymbol{=}$$

$[P\times p_{inf}\times R_{inf\_sn}\times N_{sn}]+$ *1, True positive, infectious*

$[P\times{(1-p}_{inf})\times R_{non\_inf\_sn}\times N_{sn}]+$ *2, True positive, non-infectious*

$[(1-P)\times{(1-R}_{\mathrm{sp}})\times{(1-N}_{sp})]$ *3, False positive*

Where $P$ is the prevalence of current or recent SARS-CoV-2 infection; $p_{inf}$ is the proportion amongst those tested who are in acute phase; $R_{inf\_sn}$, $R_{non\_inf\_sn}$ and $R_{sp}$is the Ag-RDT sensitivity for current infection, Ag-RDT sensitivity for recent infection, and Ag-RDT specificity, respectively, all relative to NAT; $N_{sn}$ and $N_{sp}$is NAT sensitivity and specificity, respectively.

**Probability of a negative diagnosis**

Below, are the probabilities of receiving a negative diagnosis ($D_{n\_NAT}$ and $D_{n\_RDT}$) for the different algorithms:

***NAT-based strategy***

$$\boldsymbol{D}_{\boldsymbol{n\_NAT}}=$$

$[P\times p_{NAT}\times{(1-N}_{sn})]+$ *1, False negative (NAT)*

$[P\times{(1-p}_{NAT})\times{(1-J}_{sn})]+$ *2, False negative (clinical judgement)*

$\left[ \left( 1-P \right)\times\left( 1-p_{NAT} \right)\times N_{sp} \right]+$ *3, True negative (NAT)*

$[(1-P)\times(1-p_{NAT})\times J_{sp}]$ *4, True negative (clinical judgement)*

Where $P$ is the prevalence of current or recent SARS-CoV-2 infection; $p_{NAT}$ is the proportion able to access a NAT test; $N_{sn}$ and $N_{sp}$is NAT sensitivity and specificity, respectively; $J_{sn}$and $J_{sp}$ is the sensitivity and specificity of clinical judgement in the absence of NAT, respectively.

***Ag-RDT-led strategy***

As above, the probability of a negative test result due to an Ag-RDT-led algorithm can be calculated by the following framework, with different components of the framework being zeroed by indicator variables (I_neg_ and I_pos_) depending on which algorithm is selected:

*Framework*:

$$\boldsymbol{D}_{\boldsymbol{n\_RDT}}={[D}_{n\_RDT\_only}\times{(1-I}_{neg}-I_{pos})]+[D_{n\_RDT\_neg}\times I_{neg}]+{[D}_{n\_RDT\_pos}\times I_{pos}]$$

The individual probabilities ($D_{n\_RDT\_only}$, $D_{n\_RDT\_neg}$ and $D_{n\_RDT\_pos}$) can be calculated by the following equations:

*Ag-RDT only:* ${(D}_{n\_RDT\_only})$

$$\boldsymbol{D}_{\boldsymbol{n\_RDT\_only}}=$$

$[P\times p_{inf}\times(1-R_{inf\_sn})]+$ *1, False negative, infectious*

$[P\times{(1-p}_{inf})\times{(1-R}_{non\_inf\_sn})]+$ *2, False negative, non-infectious*

$[(1-P)\times R_{sp}]$ *3, True negative*

Where $P$ is the prevalence of current or recent SARS-CoV-2 infection; $p_{inf}$ is the proportion amongst those tested who are in acute phase; $R_{inf\_sn}$, $R_{non\_inf\_sn}$ and $R_{sp}$is the Ag-RDT sensitivity for current infection, Ag-RDT sensitivity for recent infection, and Ag-RDT specificity, respectively, all relative to NAT.

*Confirmation of an Ag-RDT negative with a NAT:* $(D_{n\_RDT\_neg})$

$$\boldsymbol{D}_{\boldsymbol{n\_RDT\_neg}}=$$

$[P\times p_{inf}\times(1-R_{inf\_sn})\times p_{NAT}\times{(1-N}_{sn})]+$ *1, False negative (Ag-RDT & NAT), infectious*

$[P\times{(1-p}_{inf})\times{(1-R}_{non\_inf\_sn})\times p_{NAT}\times{(1-N}_{sn})]+$ *2, False negative (Ag-RDT & NAT), non- inf*

$[P\times p_{inf}\times(1-R_{inf\_sn})\times{(1-p}_{NAT})\times{(1-J}_{sn})]+$ *3, False negative (Ag-RDT & C.J.), infectious*

$[P\times{(1-p}_{inf})\times{(1-R}_{non\_inf\_sn})\times{(1-p}_{NAT})\times{(1-J}_{sn})]+$ *4, False negative (Ag-RDT & C.J), non-inf*

$\left[ \left( 1-P \right)\times R_{sp}\times p_{NAT}\times N_{sp} \right]+$ *5, True negative (Ag-RDT & NAT)*

$[(1-P)\times R_{sp}\times(1-p_{NAT})\times J_{sp}]$ *6, True negative (Ag-RDT & C.J)*

Where $P$ is the prevalence of current or recent SARS-CoV-2 infection; $p_{inf}$ is the proportion amongst those tested who are in acute phase; $R_{inf\_sn}$, $R_{non\_inf\_sn}$ and $R_{sp}$is the Ag-RDT sensitivity for current infection, Ag-RDT sensitivity for recent infection, and Ag-RDT specificity, respectively, all relative to NAT; $p_{NAT}$ is the proportion able to access a NAT test; $N_{sn}$ and $N_{sp}$is NAT sensitivity and specificity, respectively; $J_{sn}$and $J_{sp}$ is the sensitivity and specificity of clinical judgement in the absence of NAT, respectively.

*Confirmation of an Ag-RDT positive with a NAT:* $(D_{n\_RDT\_pos})$

$$\boldsymbol{D}_{\boldsymbol{n\_RDT\_pos}}=$$

$[P\times p_{inf}\times(1-R_{inf\_sn})]+$ *1, False negative (missed by Ag-RDT), infectious*

$[P\times{(1-p}_{inf})\times{(1-R}_{non\_inf\_sn})]+$ *2, False negative (Ag-RDT), non- infectious*

$\left[ P\times p_{inf}\times R_{inf\_sn}\times\left( 1-N_{sn} \right) \right]+$ *3, False negative (NAT), infectious*

$[P\times{(1-p}_{inf})\times R_{non\_inf\_sn}\times{(1-N}_{sn})]+$ *4, False negative (NAT), non-infectious*

$\left[ \left( 1-P \right)\times\left( 1-R_{sp} \right)\times N_{sp} \right]+$ *5, True negative (Ag-RDT & NAT)*

$[(1-P)\times R_{sp}]$  *6, True negative (Ag-RDT)*

Where $P$ is the prevalence of current or recent SARS-CoV-2 infection; $p_{inf}$ is the proportion amongst those tested who are in acute phase; $R_{inf\_sn}$, $R_{non\_inf\_sn}$ and $R_{sp}$is the Ag-RDT sensitivity for current infection, Ag-RDT sensitivity for recent infection, and Ag-RDT specificity, respectively, all relative to NAT; $N_{sn}$ and $N_{sp}$is NAT sensitivity and specificity, respectively.

**Probability of receiving a NAT test**

Here, we calculate the probability of receiving a NAT test for each algorithm.

***NAT-based strategy***

$$\boldsymbol{N}_{\boldsymbol{NAT}}{=p}_{NAT}$$

***Ag-RDT-led strategy***

As above, the probability of receiving a NAT test with an Ag-RDT-led algorithm can be calculated by the following framework, with different components of the framework being zeroed by indicator variables (I_neg_ and I_pos_) depending on which algorithm is selected:

*Framework*:

$$\boldsymbol{N}_{\boldsymbol{RDT}}={[N}_{RDT\_only}\times{(1-I}_{neg}-I_{pos})]+[N_{RDT\_neg}\times I_{neg}]+{[N}_{RDT\_pos}\times I_{pos}]$$

The individual probabilities (${(N}_{RDT\_only}$, $N_{RDT\_neg}$ and $N_{RDT\_pos}$) can be calculated by the following equations:

*Ag-RDT only:* ${(N}_{RDT\_only})$

$$\boldsymbol{N}_{\boldsymbol{RDT\_only}}\boldsymbol{=}0$$

*Confirmation of an Ag-RDT negative with a NAT:* $\left( N_{RDT\_neg} \right)$

$$\boldsymbol{N}_{\boldsymbol{RDT\_neg}}=$$

$[P\times p_{inf}\times(1-R_{inf\_sn})\times p_{NAT}]+$ *1, False negative (missed by Ag-RDT), infectious*

$[P\times{(1-p}_{inf})\times{(1-R}_{non\_inf\_sn})\times p_{NAT}]+$ *2, False negative (Ag-RDT), non- infectious*

$[(1-P)\times R_{\mathrm{sp}}\times p_{NAT}]$ *3, True negative (Ag-RDT)*

Where $P$ is the prevalence of current or recent SARS-CoV-2 infection; $p_{inf}$ is the proportion amongst those tested who are in acute phase; $R_{inf\_sn}$, $R_{non\_inf\_sn}$ and $R_{sp}$is the Ag-RDT sensitivity for current infection, Ag-RDT sensitivity for recent infection, and Ag-RDT specificity, respectively, all relative to NAT; $p_{NAT}$ is the proportion able to access a NAT test.

*Confirmation of an Ag-RDT positive with a NAT:* $(N_{RDT\_pos})$

$$\boldsymbol{N}_{\boldsymbol{RDT\_pos}}=$$

$[P\times p_{inf}\times R_{inf\_sn}]+$ *1, True positive (detected by Ag-RDT), infectious*

$[P\times{(1-p}_{inf})\times R_{non\_inf\_sn}]+$ *2, True positive (Ag-RDT), non-infectious*

$[(1-P)\times{(1-R}_{\mathrm{sp}})]$ *3, False positive (Ag-RDT)*

Where $P$ is the prevalence of current or recent SARS-CoV-2 infection; $p_{inf}$ is the proportion amongst those tested who are in acute phase; $R_{inf\_sn}$, $R_{non\_inf\_sn}$ and $R_{sp}$is the Ag-RDT sensitivity for current infection, Ag-RDT sensitivity for recent infection, and Ag-RDT specificity, respectively, all relative to NAT.

**Probability of receiving an Ag-RDT test**

Here, we calculate the probability of receiving an Ag-RDT test for each algorithm.

***NAT-based strategy***

$$\boldsymbol{R}_{\boldsymbol{NAT}}=0$$

***Ag-RDT-led strategy***

As above, the probability of receiving an Ag-RDT test with an Ag-RDT-led algorithm can be calculated by the following framework, with different components of the framework being zeroed by indicator variables (I_neg_ and I_pos_) depending on which algorithm is selected:

*Framework*:

$$\boldsymbol{R}_{\boldsymbol{RDT}}={[R}_{RDT\_only}\times{(1-I}_{neg}-I_{pos})]+[R_{RDT\_neg}\times I_{neg}]+{[R}_{RDT\_pos}\times I_{pos}]$$

The individual probabilities ($R_{RDT\_only}, R_{RDT\_neg}$ and $R_{RDT\_pos}$) can be calculated by the following equations:

*Ag-RDT only:* ${(R}_{RDT\_only})$

$$\boldsymbol{R}_{\boldsymbol{RDT\_only}}\boldsymbol{=}1$$

*Confirmation of an Ag-RDT negative with a NAT:* $\left( R_{RDT\_neg} \right)$

$$\boldsymbol{R}_{\boldsymbol{RDT\_neg}}=1$$

*Confirmation of an Ag-RDT positive with a NAT:* $(R_{RDT\_pos})$

$$\boldsymbol{R}_{\boldsymbol{RDT\_pos}}=1$$

**Probability of death due to COVID-19**

Using the probability of a true-positive test result and the probability of a false-negative test result, we calculated the probability of death due to COVID-19 for each algorithm. All the algorithms follow the same framework, listed below:

*Framework for all algorithms:*

$$probability of death=$$

$$\left[ Probability of a true positive result \times M\times M_{red} \right]+$$

$$\left[ Probability of a false negative result \times p_{treat}\times M\times M_{red} \right]+$$

$$[Probability of a false negative result \times{(1-p}_{treat})\times M]$$

Where $M$ is the case fatality rate amongst hospitalised COVID-19 patients; $M_{red}$ is the case fatality reduction amongst COVID-19 patients on dexamethasone; $p_{treat}$ is the proportion of hospitalised patients with a negative COVID-19 test result that are initiated onto dexamethasone.

**Number of infectious days per person**

Using the probability of a true-positive test result and the probability of a false-negative test result, we calculated the number of infectious days per person for each algorithm, as shown below. Note, whether individuals isolate whilst awaiting a NAT result or not can be switched on and off by the indicator variable, I_isol._

***NAT-based strategy***

$$Infectious days per person=$$

$$\left[ Probability of a true positive (NAT) \times p_{inf}\times\min\left( D_{inf},D_{NAT} \right)\times\left( 1-I_{isol} \right) \right]+$$

$$\left[ Probability of a false negative (NAT) \times p_{inf}\times\left( \left( \min\left( D_{inf},D_{NAT} \right)\times\left( 1-I_{isol} \right) \right)+\max\left( D_{inf}-D_{NAT},0 \right) \right) \right]+$$

$$[Probability of a false negative (clinical judgement){\times p}_{inf}{\times D}_{inf}]$$

Where $p_{inf}$ is the proportion amongst those tested who are in acute phase; $D_{inf}$ is the number of infectious days remaining amongst those in acute phase; $D_{NAT}$ is the NAT turnaround time.

***Ag-RDT only strategy***

$$Infectious days per person=Probability of a false negative {\times D}_{inf}$$

***Confirmation of an Ag-RDT negative with a NAT***

$$Infectious days per person=$$

$$\left[ Probability of a true positive (NAT) \times\min\left( D_{inf},D_{NAT} \right)\times\left( 1-I_{isol} \right) \right]+$$

$$\left[ Probability of a false negative (NAT)\times\left( \left( \min\left( D_{inf},D_{NAT} \right)\times\left( 1-I_{isol} \right) \right)+\max\left( D_{inf}-D_{NAT},0 \right) \right) \right]+$$

$$[Probability of a false negative (clinical judgement){\times D}_{inf}]$$

Where $D_{inf}$ is the number of infectious days remaining amongst those in acute phase; $D_{NAT}$ is the NAT turnaround time.

***Confirmation of an Ag-RDT positive with a NAT***

$$Infectious days per person=$$

$$\left[ Probability of a true positive (NAT) \times\min\left( D_{inf},D_{NAT} \right)\times\left( 1-I_{isol} \right) \right]+$$

$$\left[ Probability of a false negative (NAT)\times\left( \left( \min\left( D_{inf},D_{NAT} \right)\times\left( 1-I_{isol} \right) \right)+\max\left( D_{inf}-D_{NAT},0 \right) \right) \right]+$$

$$[Probability of a false negative (Ag RDT){\times D}_{inf}]$$

Where $D_{inf}$ is the number of infectious days remaining amongst those in acute phase; $D_{NAT}$ is the NAT turnaround time.

**Cost per person**

Here, we calculate the cost per person. The total cost of an algorithm is as follows:

***Framework for all algorithms:***

$${Cost}_{total}={Cost}_{testing}+{Cost}_{isolation}+{Cost}_{treatment}$$

Below, we breakdown the costs of the different components (testing, isolation and treatment) for each algorithm. Note, whether individuals isolate whilst awaiting a NAT result or not can be switched on and off by the indicator variable, I_isol._

***NAT-based strategy***

*Cost of testing*

$${Cost}_{NAT\_testing}=N_{NAT}\times C_{NAT}$$

Where $N_{NAT}$ is the probability of receiving a NAT test; $C_{NAT}$ is the cost per NAT test.

*Cost of isolation*

$${Cost}_{NAT\_isolation}=$$

$$\left[ p_{NAT}\times I_{isol}\times D_{NAT}\times C_{isol} \right]+$$

$$\left[ \left( D_{p\_NAT}(1)+D_{p\_NAT}(3) \right)\times I_{isol}\times\max\left( D_{isol}-D_{NAT},0 \right)\times C_{isol} \right]+$$

$$[(D_{p\_NAT}(1)+D_{p\_NAT}(3))\times{(1-I}_{isol})\times D_{isol}\times C_{isol}]+$$

$$[(D_{p\_NAT}(2)+D_{p\_NAT}(4))\times D_{isol}\times C_{isol}]$$

Where $p_{NAT}$ is the proportion able to access a NAT test; $D_{NAT}$ is the NAT turnaround time; $C_{isol}$ is the cost of isolation per person per day; $D_{p\_NAT}(1)$ and $D_{p\_NAT}(3)$ is the probability of a true-positive and a false-positive diagnosis when using a NAT, respectively; $D_{p\_NAT}(2)$ and $D_{p\_NAT}(4)$ is the probability of a true-positive and a false-positive diagnosis under clinical judgement in the absence of NAT, respectively; $D_{isol}$ is the duration of isolation.

*Cost of treatment*

$${Cost}_{NAT\_treatment}=$$

$$\left[ D_{p_{NAT}}\times D_{treat}\times C_{treat} \right]+$$

$$[D_{n_{NAT}}\times p_{treat}\times D_{treat}\times C_{treat}]$$

Where $D_{p\_NAT}$ and $D_{n\_NAT}$ is the probability of a positive and a negative diagnosis under a NAT strategy, respectively; $D_{treat}$ is the duration of treatment; $p_{treat}$ is the proportion of hospitalised patients with a negative COVID-19 test result that are initiated onto dexamethasone; $C_{treat}$ is the cost of treatment per person per day.

***Ag-RDT only***

*Cost of testing*

$${Cost}_{RDT\_testing}=C_{RDT}$$

Where $C_{RDT}$ is the cost per Ag-RDT test.

*Cost of isolation*

$${Cost}_{RDT\_isolation}=D_{p\_RDT\_only}\times D_{isol}\times C_{isol}$$

Where $C_{isol}$ is the cost of isolation per person per day; $D_{p\_RDT\_only}$ is the probability of a positive diagnosis under an Ag-RDT only strategy; $D_{isol}$ is the duration of isolation.

*Cost of treatment*

$${Cost}_{RDT\_treatment}=$$

$$\left[ D_{p_{RDTonly}}\times D_{treat}\times C_{treat} \right]+$$

$$[D_{n_{RDTonly}}\times p_{treat}\times D_{treat}\times C_{treat}]$$

Where $D_{p_{RDTonly}}$ and $D_{n_{RDTonly}}$ is the probability of a positive and a negative diagnosis under an Ag-RDT only strategy, respectively; $D_{treat}$ is the duration of treatment; $p_{treat}$ is the proportion of hospitalised patients with a negative COVID-19 test result that are initiated onto dexamethasone; $C_{treat}$ is the cost of treatment per person per day.

***Confirmation of an Ag-RDT negative with a NAT***

*Cost of testing*

$${Cost}_{RDT\_neg\_testing}=C_{RDT}+[N_{RDT\_neg}\times C_{NAT}]$$

Where $C_{RDT}$ is the cost per Ag-RDT test; $C_{NAT}$ is the cost per NAT test; $N_{RDT\_neg}$ is the probability of receiving a NAT test under a confirm Ag-RDT negative strategy.

*Cost of isolation*

$${Cost}_{RDT\_neg\_isolation}=$$

$$\left[ \left( D_{p\_RDT\_neg}(1)+D_{p\_RDT\_neg}(2)+D_{p\_RDT\_neg}(7)+D_{p\_RDT\_neg}(5)+D_{p\_RDT\_neg}(6)+D_{p\_RDT\_neg}(9) \right)\times D_{isol}\times C_{isol} \right]+$$

$$\left[ N_{RDT\_neg}\times I_{isol}\times D_{NAT}\times C_{isol} \right]+$$

$$\left[ \left( D_{p\_RDT\_neg}(3)+D_{p\_RDT\_neg}(4)+D_{p\_RDT\_neg}(8) \right)\times I_{isol}\times\max\left( D_{isol}-D_{NAT},0 \right)\times C_{isol} \right]+$$

$$[\left( D_{p\_RDT\_neg}(3)+D_{p\_RDT\_neg}(4)+D_{p\_RDT\_neg}(8) \right)\times{(1-I}_{isol})\times D_{isol}\times C_{isol}]$$

Where $D_{p\_RDT\_neg}(1)$ and $D_{p\_RDT\_neg}(2)$ is the probability of a true positive diagnosis when using an Ag-RDT amongst infectious and non-infectious cases, respectively; $D_{p\_RDT\_neg}(5)$ and $D_{p\_RDT\_neg}(6)$ is the probability of a true positive diagnosis when using clinical judgment in the absence of NAT amongst infectious and non-infectious cases, respectively; $D_{p\_RDT\_neg}(7)$ and $D_{p\_RDT\_neg}(9)$ is the probability of a false positive diagnosis when using an Ag-RDT or under clinical judgement in the absence of NAT, respectively; $D_{p\_RDT\_neg}(3)$ and $D_{p\_RDT\_neg}(4)$ is the probability of a true positive diagnosis when using NAT amongst infectious and non-infectious cases, respectively; $D_{p_{RDT_{neg}}}(8)$ is the probability of a false positive diagnosis when using NAT; $N_{RDT\_neg}$ is the probability of receiving a NAT test; $C_{isol}$ is the cost of isolation per person per day; $D_{isol}$ is the duration of isolation; $D_{NAT}$ is the NAT turnaround time.

*Cost of treatment*

$${Cost}_{RDT\_neg\_treatment}=$$

$$\left[ D_{p_{RDTneg}}\times D_{treat}\times C_{treat} \right]+$$

$$[D_{n\_RDTneg}\times p_{treat}\times D_{treat}\times C_{treat}]$$

Where $D_{p_{RDTneg}}$ and $D_{n\_RDTneg}$ is the probability of a positive and a negative diagnosis under a confirm Ag-RDT negative strategy, respectively; $D_{treat}$ is the duration of treatment; $p_{treat}$ is the proportion of hospitalised patients with a negative COVID-19 test result that are initiated onto dexamethasone; $C_{treat}$ is the cost of treatment per person per day.

***Confirmation of an Ag-RDT positive with a NAT***

*Cost of testing*

$${Cost}_{RDT\_pos\_testing}=C_{RDT}+[N_{RDT\_pos}\times C_{NAT}]$$

Where $C_{RDT}$ is the cost per Ag-RDT test; $C_{NAT}$ is the cost per NAT test; $N_{RDT\_pos}$ is the probability of receiving a NAT test under a confirm Ag-RDT positive strategy.

*Cost of isolation*

$${Cost}_{RDT\_pos\_isolation}=$$

$$\left[ N_{RDT\_pos}\times I_{isol}\times D_{NAT}\times C_{isol} \right]+$$

$$\left[ D_{p\_RDT\_pos}\times I_{isol}\times\max\left( D_{isol}-D_{NAT},0 \right)\times C_{isol} \right]+$$

$$[D_{p\_RDT\_pos}\times{(1-I}_{isol})\times D_{isol}\times C_{isol}]$$

Where $D_{p\_RDT\_pos}$ is the probability of a positive diagnosis; $N_{RDT\_pos}$ is the probability of receiving a NAT test; $C_{isol}$ is the cost of isolation per person per day; $D_{isol}$ is the duration of isolation; $D_{NAT}$ is the NAT turnaround time.

*Cost of treatment*

$${Cost}_{RDT\_pos\_treatment}=$$

$$\left[ D_{p_{RDTpos}}\times D_{treat}\times C_{treat} \right]+$$

$$[D_{n\_RDTpos}\times p_{treat}\times D_{treat}\times C_{treat}]$$

Where $D_{p_{RDTpos}}$ and $D_{n\_RDTpos}$ is the probability of a positive and a negative diagnosis under a confirm Ag-RDT positive strategy, respectively; $D_{treat}$ is the duration of treatment; $p_{treat}$ is the proportion of hospitalised patients with a negative COVID-19 test result that are initiated onto dexamethasone; $C_{treat}$ is the cost of treatment per person per day.
