# Supplementary figures and images for: Quantifying the potential value of antigen-detection rapid diagnostic tests for COVID-19: a modelling analysis
Source: BMC Med. 2021 Mar 9;19:75. doi: 10.1186/s12916-021-01948-z (PMC7939929; doi:10.1186/s12916-021-01948-z)

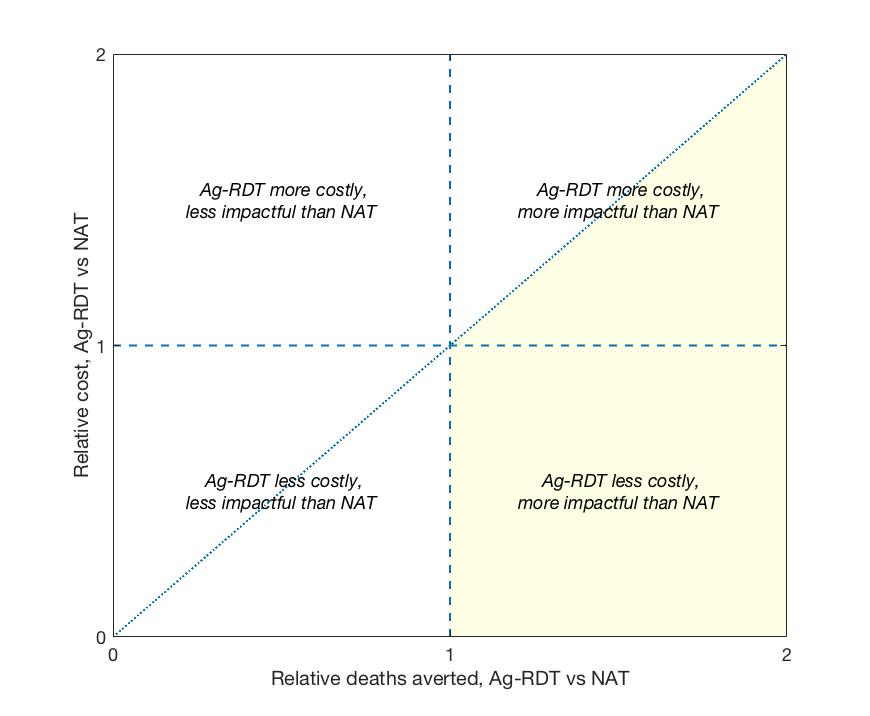

Supplement: Supplementary file 3 — Additional file 3: Figure S1. Schematic illustration for visualising the value of an Ag-RDT-led strategy, relative to a scenario involving NAT and clinical judgement. Although the figure involves deaths averted, the same structure applies for averting infectious person-days. For a given set of parameters drawn from the parameter ranges shown in Table 2, we simulated the cost and impact of a given Ag-RDT-led strategy, and of a NAT-based testing strategy, both relative to a no-intervention scenario. This outcome was then represented in the figure by plotting the relative deaths averted by Ag-RDT vs NAT (horizontal axis) against the relative cost of the two strategies (vertical axis). Thus, for example, in the lower right quadrant, an Ag-RDT-led strategy would cost less, but have more impact, than NAT. The diagonal dashed line shows an important threshold: for points below this line, an Ag-RDT-led strategy would cost less per death averted than NAT, and vice versa. Overall, therefore, the shaded area shows the region in which an Ag-RDT would simultaneously cost less per death averted, and avert more deaths overall, than NAT. We denote this area as the ‘favourable region’ for an Ag-RDT, and elsewhere as ‘non-favourable’: in our current analysis we aim to identify the circumstances under which an Ag-RDT, of a given performance and cost, would occupy this region. [file 12916_2021_1948_MOESM3_ESM.jpg]

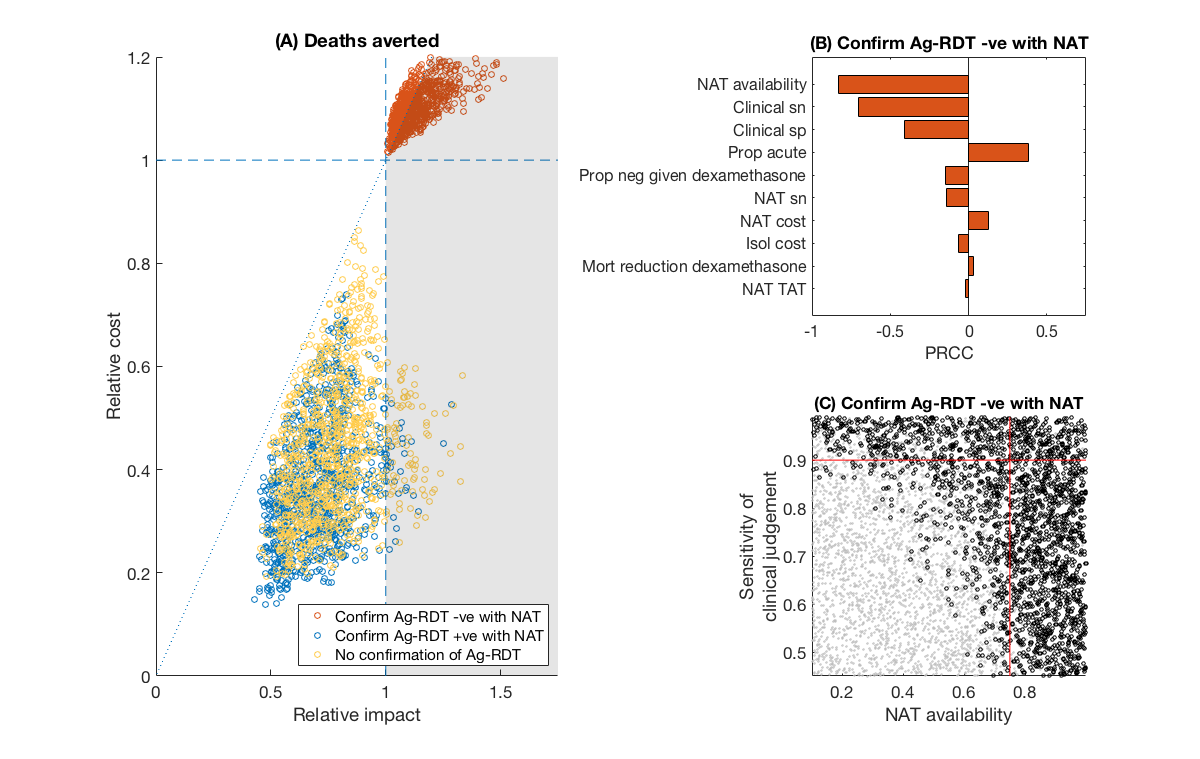

Supplement: Supplementary file 5 — Additional file 5: Figure S2. Relative value of Ag-RDT-led vs NAT-based testing, for averting deaths in a hospital setting. The figure shows the same results as those presented in Fig. 2 in the main text, but here assuming that all patients awaiting a NAT result (whether as part of a NAT-based strategy or for confirmation of Ag-RDT results) were not isolated during this time. Results illustrate qualitatively similar findings to those shown in the main text. In panel (A), in the scenario where Ag-RDT-negative results were confirmed using NAT (red points), 57% of simulations placed the Ag-RDT-led strategy in the favourable region, below the diagonal dashed line. Panels (B, C) show additional sensitivity analyses for these points in particular, as described in Fig. 2. In (C), red lines show 75% NAT availability (vertical line), and 90% sensitivity of clinical judgement (horizontal line). In the lower left quadrant of these lines, an Ag-RDT was favourable over NAT in 85% of simulations. [file 12916_2021_1948_MOESM5_ESM.tif]

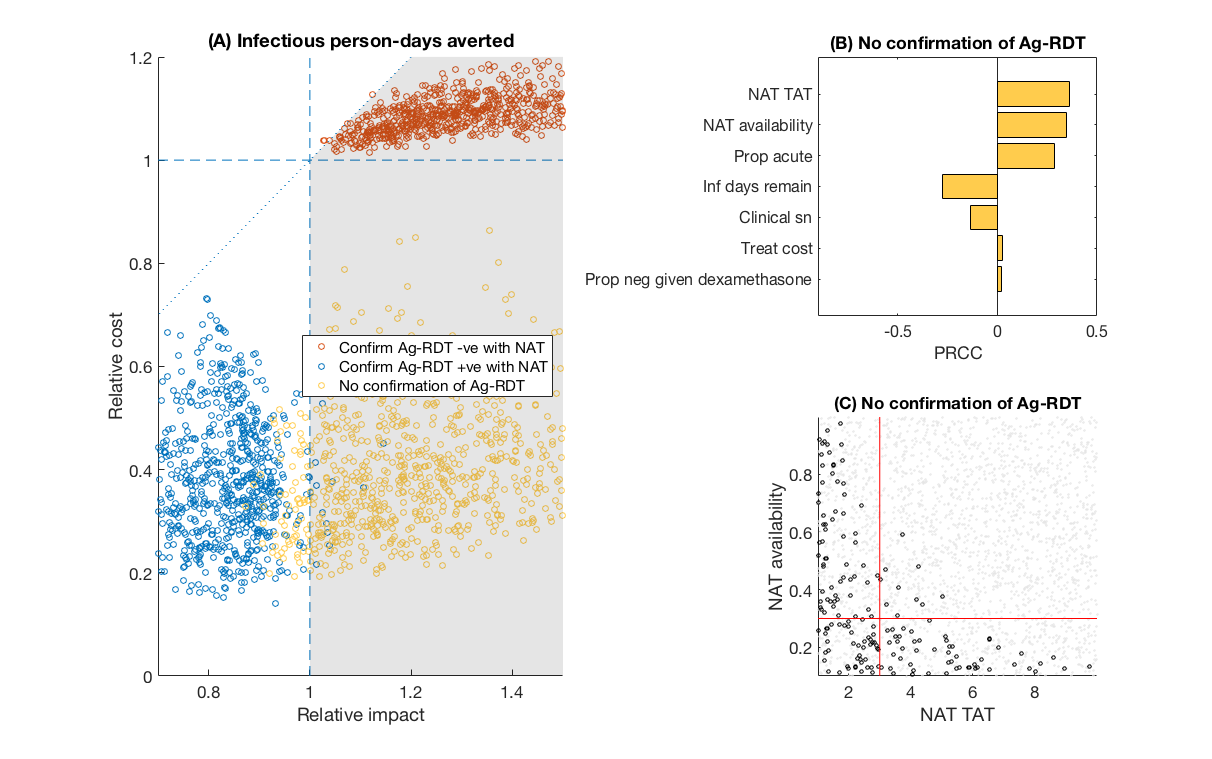

Supplement: Supplementary file 6 — Additional file 6: Figure S3. Relative value of Ag-RDT-led vs NAT-based testing, for averting infections in a hospital setting. The figure shows the same results as those presented in Fig. 3 in the main text, but here assuming that all patients awaiting a NAT result (whether as part of a NAT-based strategy or for confirmation of Ag-RDT results) were not isolated during this time. In panel (A), in the scenario where there was no NAT confirmation of Ag-RDT results (yellow points), 93% of simulations placed the Ag-RDT-led strategy in the favourable region, to the right of the vertical, dashed line. Panels (B, C) show additional sensitivity analyses for these points in particular, as described in Fig. 3. In (C), red lines show a NAT turnaround time of 3 days (vertical line), and a 30% NAT availability (horizontal line). In the upper right quadrant of these lines, an Ag-RDT was favourable over NAT in 69% of simulations. [file 12916_2021_1948_MOESM6_ESM.tif]

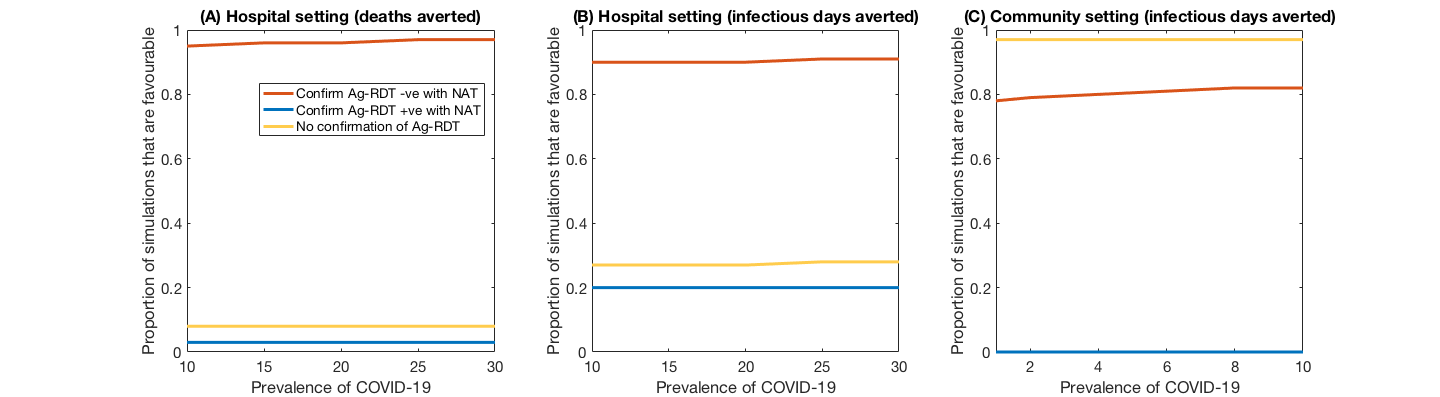

Supplement: Supplementary file 7 — Additional file 7: Figure S4. Sensitivity analysis to varying prevalence of COVID-19 amongst those being tested. As a focal model output, all figures show the proportion of simulations in which an Ag-RDT was favourable, with different algorithms labelled by the different line colours. Panels A and B show the impact of varying prevalence on deaths and infectious days averted, respectively, in a hospital setting. Panel C shows the impact on infectious days averted in a community setting. Similar to the analysis presented in the main text, we assumed that all individuals were isolated whilst waiting for a NAT result in the hospital setting and that no one isolated whilst awaiting a test result in the community setting. Results illustrate that the proportion favourable remained stable to these alternative assumptions for prevalence. [file 12916_2021_1948_MOESM7_ESM.tif]

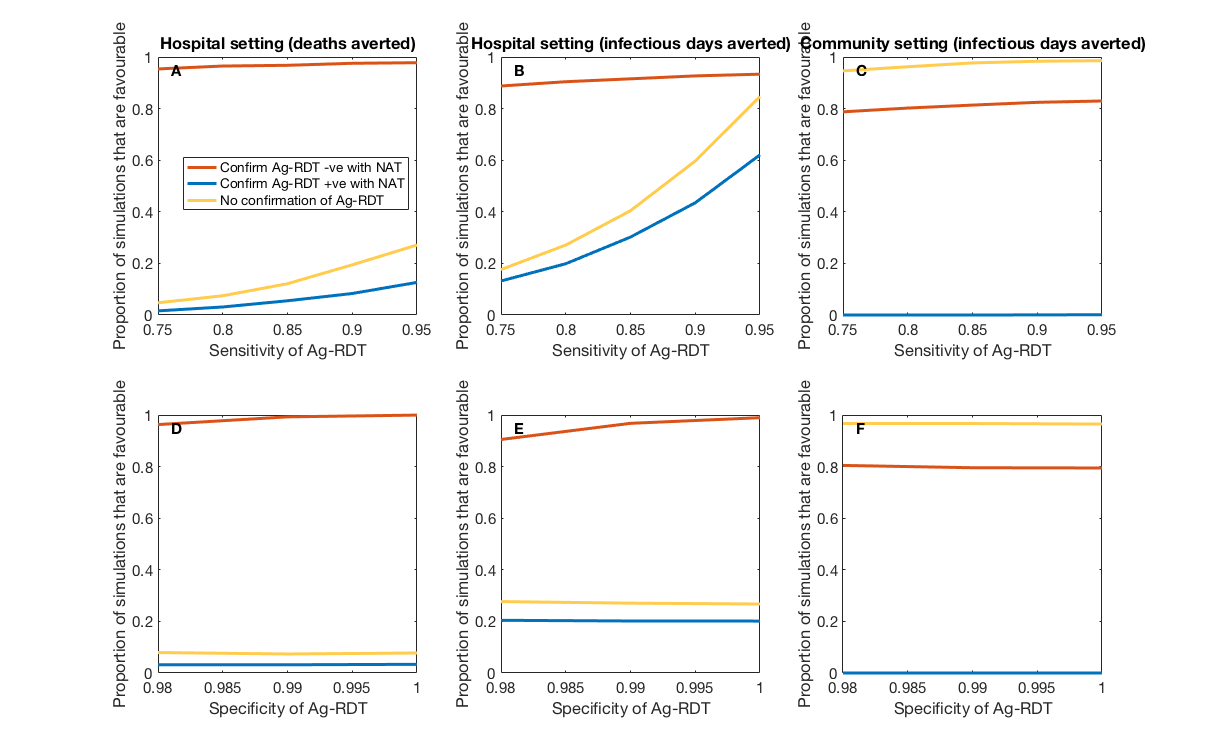

Supplement: Supplementary file 8 — Additional file 8: Figure S5. Sensitivity analysis to varying Ag-RDT sensitivity and specificity. As a focal model output, all figures show the proportion of simulations in which an Ag-RDT was favourable, with different algorithms labelled by the different line colours. Panels A-C show the sensitivity of Ag-RDT being varied between 75 and 95% across the hospital and community settings, assuming specificity remained fixed at 98%. Panels D-F show the specificity of Ag-RDT being varied between 98 and 100%, assuming sensitivity remained fixed at 80%. Similar to the analysis presented in the main text, we assumed that all individuals were isolated whilst waiting for a NAT result in the hospital setting and that no one isolated whilst awaiting a test result in the community setting. Results illustrate that, in a community setting, increasing Ag-RDT sensitivity increased the favourability of the “Ag-RDT only” and “confirm Ag-RDT negative” strategies (panel C). For example, the favourability of an algorithm that confirms an Ag-RDT negative result increased from 79% to 83% when sensitivity increased from 75% to 95%. Increasing sensitivity had little impact on the “confirm Ag-RDT positive” strategy; since the only costs incurred under a community setting was the cost of a test, a NAT-only strategy was often cheaper and averted more infectious days than the “confirm Ag-RDT positive” strategy (the cost of testing with an Ag-RDT and confirming a positive result with a NAT test makes it costly, and by re-testing a positive result with a NAT, the sensitivity of the algorithm was lower due to the imperfect sensitivity of NAT). Similar to the hospital setting, specificity had little impact on an algorithm’s favourability (panel F). [file 12916_2021_1948_MOESM8_ESM.tif]

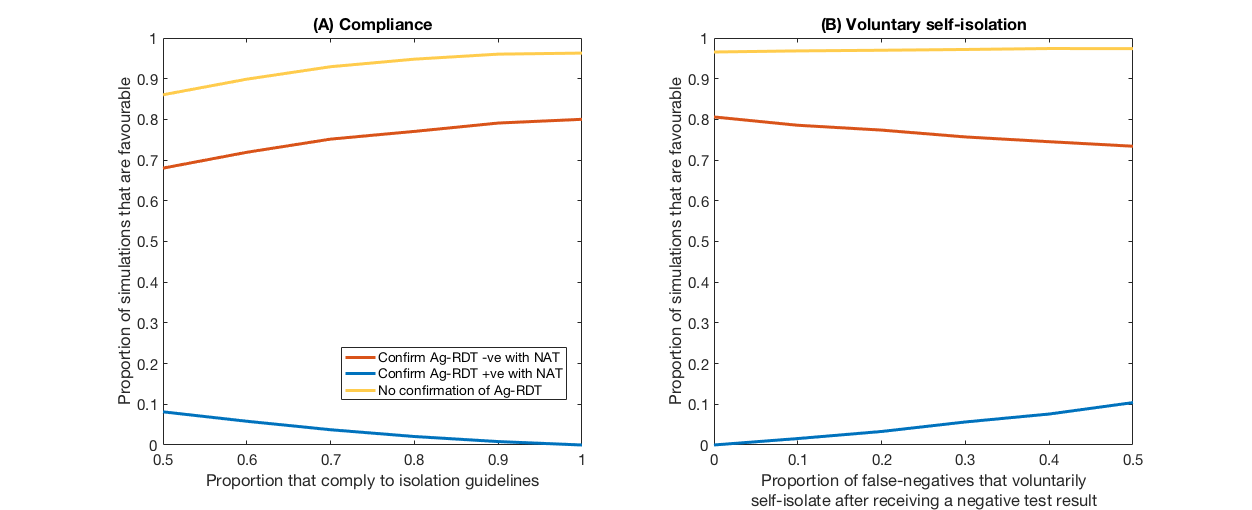

Supplement: Supplementary file 9 — Additional file 9: Figure S6. Sensitivity analysis to patient behaviour in relation to self-isolation, in the community setting. As a focal model output, all figures show the proportion of simulations in which an Ag-RDT was favourable, with different algorithms labelled by the different line colours. Panel A shows the impact of compliance amongst those required to self-isolate after a positive final test result. Panel B shows the impact of test-negative individuals voluntarily self-isolating. This sensitivity analysis was restricted to the community setting as it is likely that hospitals will enforce compliance to isolation guidelines. Results illustrate that increasing the proportion of compliance to isolation recommendations increased the favourability of both “Ag-RDT-only” and “confirm Ag-RDT negative” strategies, from 86% and 68% of simulations being favourable with 50% compliance to 98% and 80% with 100% compliance, respectively. The benefit of an Ag-RDT test in rapidly detecting COVID cases, and hence averting onward transmission, is reduced if these individuals did not isolate. However, the opposite was seen with the “confirm Ag-RDT positive” strategy, with the favourability of the algorithm decreasing from 8% to 0% if compliance doubled from 50% to 100%. Generally, this strategy detected fewer COVID cases than a NAT-based strategy, due to the reduction in overall sensitivity caused by inclusion of NAT confirmation; thus, increasing the proportion of individuals that did comply had a greater effect on a NAT-based strategy than the Ag-RDT strategy, hence increasing the latter’s favourability. Similar results were seen for voluntary self-isolation (where false negatives voluntarily self-isolate). [file 12916_2021_1948_MOESM9_ESM.tif]
