# Supplementary material for: Quantifying the potential value of antigen-detection rapid diagnostic tests for COVID-19: a modelling analysis
Source: BMC Med. 2021 Mar 9;19:75. doi: 10.1186/s12916-021-01948-z (PMC7939929; doi:10.1186/s12916-021-01948-z)
Supplement: Supplementary file 4 — Additional file 4: Table S1. Summary of cost per death or infectious person-day averted of results presented in the main text. [file 12916_2021_1948_MOESM4_ESM.docx]

**Additional file 3: Table S1**

| Testing strategy | | Hospital setting | | Community setting |
| --- | --- | --- | --- | --- |
|  |  | **Cost per death averted ($)** | **Cost per infectious person-day isolated ($)** | **Cost per infectious person-day isolated ($)** |
| NAT-based | | $150,000  (38,000-490,000) | $560 (150-1,600) | $84 (11-670) |
| Ag-RDT-led | No Ag-RDT confirmation | $53,000  (14,000-150,000) | $160 (42-440) | $12 (8-23) |
|  | Confirm Ag-RDT -ve with NAT | $140,000  (36,000-440,000) | $530 (140-1,500) | $58 (17-190) |
|  | Confirm Ag-RDT +ve with NAT | $54,000  (15,000-160,000) | $150 (42-420) | $51 (18-790) |

**Table S1. Summary of cost per death or infectious person-day averted of results presented in the main text**. As explained in the Methods, impact and cost are estimated relative to a baseline of no testing, and no intervention. Numbers in brackets give 95% uncertainty intervals.
